# The origin and diversification of Amaryllidaceae: A phylogenetic and biogeographic analysis

**DOI:** 10.1002/ajb2.70092

**Published:** 2025-09-11

**Authors:** Zoë H. Dennehy‐Carr, Kálmán Könyves, Chris Yesson, John C. David, Alastair Culham

**Affiliations:** ^1^ Herbarium, School of Biological Sciences, University of Reading Reading, Berkshire RG6 6EX UK; ^2^ Florida Museum of Natural History University of Florida Gainesville 32611 Florida USA; ^3^ Royal Horticultural Society, RHS Garden Wisley, Woking Surrey GU23 6QB UK; ^4^ Zoological Society of London Regents Park London NW1 4RY UK

**Keywords:** Amaryllidaceae, biogeography, divergence times, geophyte, molecular phylogeny, monocot evolution, plastid DNA

## Abstract

**Premise:**

Previous angiosperm‐wide studies estimated that the geophytic family Amaryllidaceae diverged in Africa 87.00–46.77 million years ago (mya), spanning the Cretaceous and Palaeogene periods, including multiple important climatic and geological events. Greater precision on when and where this divergence occurred is lacking due to limited sampling of Amaryllidaceae and the paucity of the monocot fossil record. A robust phylogeny is required to estimate the age and origin of suprageneric groups; however, the evolutionary relationships within Amaryllidaceae are unclear.

**Methods:**

We used 78 plastome protein‐coding genes to infer the phylogenetic relationships of Amaryllidaceae and estimated the age of the family using four fossils and five secondary calibration points from across the Asparagales. We conducted a new biogeographic analysis to determine the ancestral origins of Amaryllidaceae and suprageneric groups, providing insights into the drivers of diversification.

**Results:**

Our phylogenetic analyses recovered Amaryllidaceae as monophyletic, with Agapanthoideae sister to Amaryllidoideae and Allioideae. We estimate that Amaryllidaceae diverged in southern Africa 48.6 mya (50.3–46.6 mya) during the early Eocene, a period of elevated global temperatures with increasing seasonal aridity. Our biogeographic analyses indicate that taxa migrated from Africa via the Arabian Peninsula to temperate Asia and beyond during the Miocene.

**Conclusions:**

The comprehensive taxon sampling across Amaryllidaceae, the greater number of genes, and the placement of fossils has made it possible to substantially refine estimates of lineage divergence. Establishing a robust age estimate and reconstructing the biogeographic history has led to a better understanding of evolution within the family, of present‐day distributions, and of possible drivers of diversification.

Global distribution and diversification into a wide range of habitats can be better understood through well‐sampled and dated phylogenetic studies. Amaryllidaceae J.St‐Hil. *sensu* APG III (2009) is a predominantly bulbous geophytic family with some rhizomatous genera (*Agapanthus* L'Hér, *Clivia* Lindl., *Cryptostephanus* Welw. *ex* Baker, and *Scadoxus* Raf.), identified by scapose and umbellate inflorescences, with bracts enclosing the flower buds (Meerow and Snijman, 1998; APG II, 2003; Chase et al., 2009; Simpson, 2010) (Figure 1). The family has a cosmopolitan distribution, with centers of diversity in the Mediterranean Basin, South Africa, and the South American tropics, as well as temperate regions of the Northern Hemisphere. Species occur in a range of climates and habitats, despite life forms and cycles being moderately consistent across the family. Understanding the biogeographic history of this family will enhance our understanding of dispersal and niche evolution of other angiosperm lineages with estimated ancestral origins in Africa, following the separation of Gondwana.

**Figure 1:**
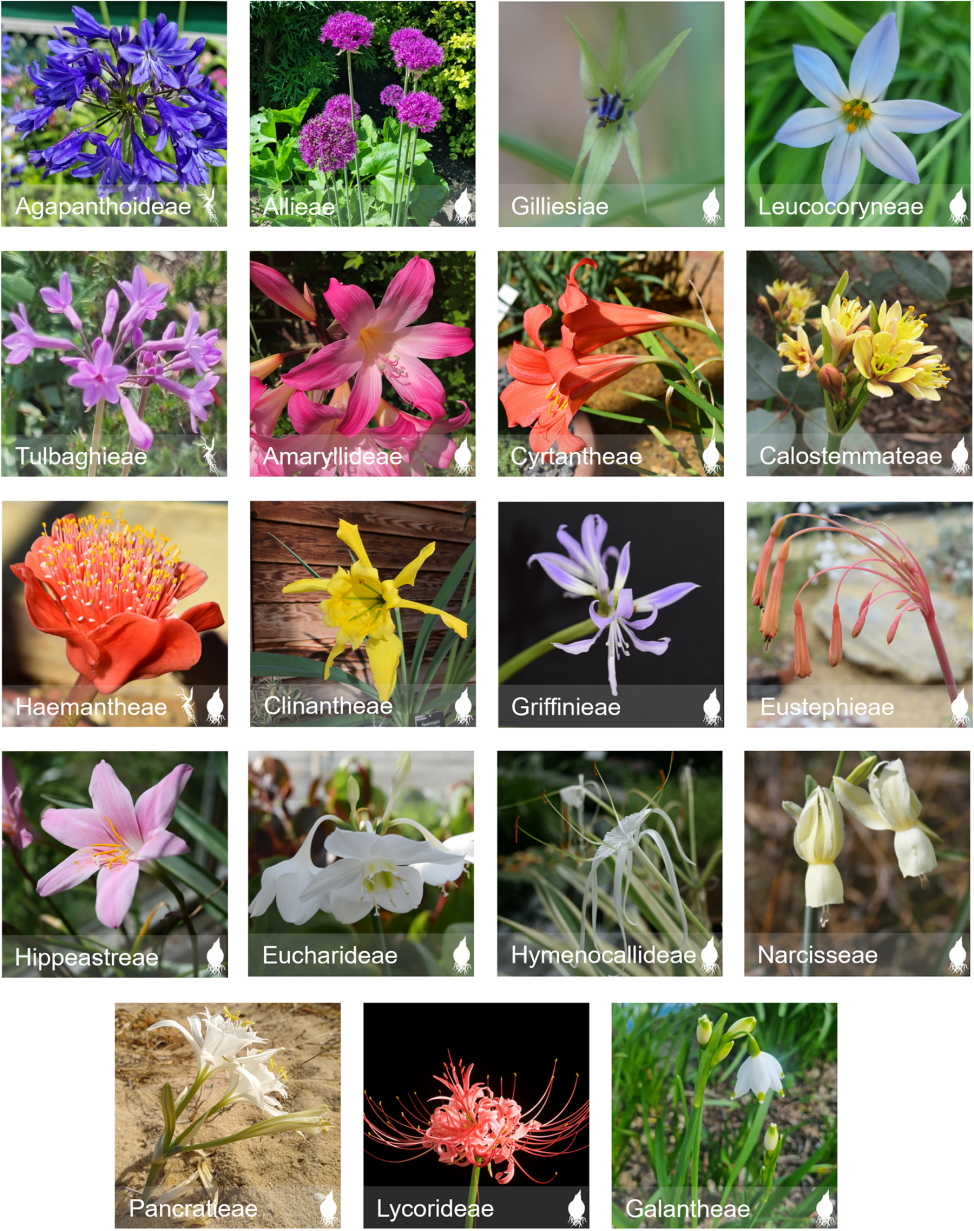
Members of each Amaryllidaceae tribe, showing examples of morphological variation across the family. Photos belong to Zoë Dennehy‐Carr, Alastair Culham, and John David, except for Calostemmateae (Melburnian, 2012). Geophytic habit(s) are represented for each tribe (bulb = 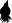; rhizome = 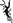).

The family comprises three subfamilies: Agapanthoideae Endl., Allioideae Herb., and Amaryllidoideae Burnett, with approximately 70 genera and 1700–1800 species (Meerow, 2023; POWO, 2024). Subfamily Agapanthoideae is monogeneric (*Agapanthus*), characterized by superior ovaries, distinguishing the genus from members of Amaryllidoideae, and lack the distinctive alliaceous (onion‐like) odor of members of Allioideae (Snoeijer, 2004; Zhang et al., 2010). The combination of a rhizome and blue flowers is also distinctive of the subfamily. Allioideae is subdivided into four tribes: Allieae (one genus, *Allium*), Gilliesieae (eight genera), Leucocoryneae (seven genera), and Tulbaghieae (one genus, *Tulbaghia* L.), following Sassone et al. (2014) and García et al. (2022a, 2022b). Members of subfamily Allioideae are distinguished by their distinctive alliaceous odor, superior ovaries, a hollow style, and spirally arranged leaves (Fay and Chase, 1996; Simpson, 2010; Meerow, 2023). Amaryllioideae taxa can be separated from Allioideae and Agapanthoideae by the presence of inferior ovaries (Simpson, 2010; Meerow et al., 2020). Both Amaryllidoideae and Agapanthoideae have solid styles and distichous leaves (Fay and Chase, 1996). The Amaryllidoideae subfamily has 14 tribes: Amaryllideae, Calostemmateae, Cyrtantheae, Haemantheae, Eurasian tribes (Galantheae, Lycorideae, Narcisseae, and Pancratieae), and American tribes (Griffineae, Hippeastreae, Clinantheae, Eucharideae, Eustephieae, Hymenocallideae) (Meerow, 2023). The relationships between several Amaryllidoideae groups have remained unresolved by previous molecular phylogenetic studies, leading to an unclear understanding of evolutionary relationships and continued recognition of polyphyletic genera (Meerow et al., 1999; Meerow and Snijman, 2006; Rønsted et al., 2012). Current tribal concepts for the Eurasian and American tribes of Amaryllidoideae are only partially supported by previous phylogenetic research (Ito et al., 1999; Rønsted et al., 2012; García et al., 2014, 2017; Meerow et al., 1999, 2000, 2006, 2020; Zuntini et al., 2024). This led to the informal naming of two monophyletic groups: the American clade (including all American tribes) and the Eurasian clade (including all Eurasian tribes) (*sensu* Meerow et al., 1999). In the present study, tribal and suprageneric groups listed follow those described in the review by Meerow (2023), recognizing four Amaryllidoideae tribes (Amaryllideae, Cyrtantheae, Calostemmateae, and Haemantheae), the American clade, and the Eurasian clade (Appendix S1).

Crown divergence time estimates for Amaryllidaceae of 87.00–46.77 mya, are derived from broader angiosperm and monocotyledon studies that have limited representation of Amaryllidaceae taxa (Magallón et al., 2015; Givnish et al., 2018), do not include all subfamilies or tribes (Chen et al., 2013; Magallón et al., 2015; Givnish et al., 2018), or have incongruent topologies related to taxon‐specific phylogenetic research (Janssen and Bremer, 2004; Zuntini et al., 2024), reducing the certainty of divergence dates. These estimates of divergence span multiple important climatic and geological events in earth's history, including the Cretaceous–Tertiary extinction event (66 mya; Westerhold et al., 2020), the Paleocene‐Eocene Thermal Maximum (56 mya; Westerhold et al., 2020), and the collision of the Indian subcontinent with Eurasia (55–40 mya; Wu et al., 2023). Additionally, a biogeographic analysis has not been conducted for the entirety of Amaryllidaceae; previous biogeographic reconstructions of Amaryllidaceae either were conducted without a dated phylogeny (Ito et al., 1999; Meerow et al., 1999); were focused on suprageneric groups including Allioideae (Costa et al., 2020; Namgung et al., 2021), Amaryllidoideae tribes (Meerow et al., 2020); or were focused on genera such as *Narcissus* L. (Santos‐Gally et al., 2012). The results of these studies proposed an African origin for subfamilies Amaryllidoideae (Ito et al., 1999; Meerow et al., 1999) and Allioideae (Costa et al., 2020; Namgung et al., 2021); however, hypotheses on the dispersal of Amaryllidaceae lineages beyond Africa differ between these studies. As a result, the biogeographic history of the family and the evolutionary drivers that caused taxa to diverge remain unclear. Reconstructing the biogeographic history of Amaryllidaceae can enhance our understanding of the evolution across the family and deepen our understanding of present‐day distributions.

Here, we construct a plastome phylogeny of Amaryllidaceae, using 78 plastome protein‐coding genes for 162 Amaryllidaceae samples encompassing all three subfamilies, all tribes, 52 genera, and 150 species. We acquire 96 samples from previous studies and include 66 newly sequenced whole plastomes. The significant increase in plastome markers and taxonomic coverage compared with previous phylogenetic studies will provide the most comprehensive understanding of plastome evolutionary relationships of Amaryllidaceae and clarify previously unresolved relationships at subfamilial, tribal, and generic levels. To date the divergence of Amaryllidaceae, we relied on fossils and secondary dates across Asparagales and used plastid protein‐coding genes from the dated Monocotyledon alignment by Givnish et al. (2018). This has allowed us to more confidently estimate the divergence of Amaryllidaceae, its subfamilies, and previously recognized groups (Meerow et al., 1999; Chase et al., 2009). Our biogeographic analysis will determine the ancestral origin of the family and suprageneric groups, identifying drivers of dispersal and diversification into a range of climates and habitats.

## MATERIALS AND METHODS

### Plant materials and taxon sampling

DNA was extracted from silica‐dried plant material for 66 samples representing 65 Amaryllidaceae taxa, covering 37 genera and all subfamilies (Appendix S2), using the Qiagen Dneasy Plant Mini Kit (Qiagen, Manchester, UK) following the manufacturer's protocol (Qiagen, 2016). DNA content was quantified using gel electrophoresis and a Qubit Fluorometer (Thermo Fisher Scientific, Altrincham, UK).

### Plastome assembly, alignment, and annotation

Whole genomic DNA extractions were sent to a commercial service for sequencing. Library development and 150 bp paired‐end (PE) sequencing for genome skimming were conducted by Novogene Corporation (Cambridge, UK) and Daicel Arbor Biosciences (Ann Arbor, Michigan, USA). This service returned fastq sequence files for analysis. Plastome assembly was conducted with Fast‐Plast version 1.2.6 (McKain and Wilson, 2017), NOVOPlasty version 2.7.0 (Dierckxsens et al., 2017), and GetOrganelle version 1.7.5 (Jin et al., 2020). The bowtie reference indices for Fast‐Plast were built using included Asparagales plastomes. The default seed of GetOrganelle was used for plastome assembly. The starting seed for NOVOPlasty assemblies was the complete plastome of *Narcissus poeticus* L. (MH706763; Kӧnyves et al., 2018). Differences between assemblies were mapped to reference in Geneious Prime version 2022.0.2 (https://www.geneious.com/) to determine the most supported assembly based on a consensus between assemblies. Complete plastomes were annotated against the same *N. poeticus* plastome in Geneious Prime. Start and stop codons of protein‐coding genes were checked against the *N. poeticus* plastome. The GenBank and SRA accession codes can be found in Appendix S2.

### GenBank and SRA data

Forty‐one Amaryllidaceae plastomes and a further four Asparagales plastomes were downloaded from GenBank (Appendix S3). Sequence read archive (SRA) data were obtained from García et al. (2014) and Meerow et al. (2020) and assembled using the custom loci approach of GetOrganelle version 1.7.5 (Jin et al., 2020) and mapped to the *N. poeticus* plastome in Geneious Prime. The plastome protein‐coding genes of eight species of the American clade generated in this study were used for the starting seed file (Appendix S4). Recovered plastid genes that had missing start or stop codons compared to the reference, that had ambiguous base pairs in the start and/or stop codons, or that were putative pseudogenes were discarded. The criteria of Joyce et al. (2018) were used to define putative pseudogenes, in which plastid protein‐coding genes with internal or missing stop codons and unclear start codons were classified as putative pseudogenes.

### Phylogenetic analysis

Protein‐encoding regions for a total of 78 plastid genes were compiled for 162 sequenced samples (66 newly sequenced accessions, 41 from GenBank, and 55 from SRA data) and four outgroups: *Asparagus officinalis* L. (KY346194), *Cordyline indivisa* (G.Forst.) Endl. (KX822776), *Hyacinthoides non‐scripta* (L.) Chouard *ex* Rothm. (MN824434; Garnett et al., 2020), and *Xanthorrhoea preissii* Endl. (KX822774). The plastome protein‐coding regions were aligned using Muscle version 3.8 (Edgar, 2004) for eight iterations. Each gene was trimmed to exclude poorly aligned regions using TrimAl version 1.2 (Capella‐Gutiérrez et al., 2009) with the “automated1” option, which automatically selects the best method to trim the multiple sequence alignment. The *cemA* gene was missing or suspected to be a pseudogene in 110 of 162 samples and subsequently excluded from further analyses. Trimmed gene alignments were concatenated using Geneious Prime, resulting in a total alignment length of 67,726 bp. ModelFinder implemented in IQ‐TREE version 2.2 (Minh et al., 2020) was used to determine the best available evolutionary model (Kalyaanamoorthy et al., 2017) for the concatenated alignment.

Phylogenetic reconstruction was conducted using maximum likelihood and Bayesian inference methods. Both methods were used to allow the assessment of any topological differences that might occur due to differing methods of estimating phylogenetic relationships (Baum and Smith, 2013). Maximum likelihood estimation was conducted in IQ‐TREE version 2.2 (Minh et al., 2020) using 1000 ultrafast bootstrap replicates (Hoang et al., 2018). Bayesian inference was conducted using MrBayes version 3.2.7a (Ronquist et al., 2012) with two Markov chain Monte Carlo runs, each with four chains. Sampling was conducted every 10,000 generations, to minimize autocorrelation, for a total of 20 million generations. Convergence was assessed using Tracer version 1.7.2 (Rambaut et al., 2018). The first 25% of trees were discarded as burn‐in to ensure that convergence had been reached and trees from the remaining generations were used to construct a consensus tree with posterior probabilities.

The number of plastome protein‐coding genes for each sample ranged from 49 to 78 due to missing data, gene losses, and pseudogenes. To explore the impact of missing data, we compared phylogenetic trees constructed using only samples with 75–78 plastome protein‐coding genes (ensuring that all subfamilies and Amaryllidoideae tribes were represented) and all samples of this study (49–78 plastome protein‐coding genes). The methods of sequence alignment construction and maximum likelihood phylogenetic analysis were the same as that of the full analysis.

### Divergence dating

We estimated divergence dates for Amaryllidaceae, all subfamilies, and widely recognized suprageneric groups using MrBayes version 3.2.7a (Ronquist et al., 2012) including seventy‐three Amaryllidaceae samples and 18 further samples from the Asparagales order to allow for the placement of fossils and secondary calibration points (Appendices S5 and S6). The number of Amaryllidaceae taxa included in this analysis was reduced to decrease the effect of missing data and the size of the data set, subsequently enhancing the signal‐to‐noise ratio and reducing the time needed to sufficiently explore tree space (Sanderson and Shaffer, 2002; Sanderson et al., 2011; Roure et al., 2013). To achieve this, all SRA data were excluded from the analysis due to the high levels of missing data and the number of samples from each genus was reduced to two, with the most distantly related samples retained for each genus. This ensured that the same branching patterns between genera were recovered as in the full Amaryllidaceae phylogenetic analysis. In the case of polyphyletic genera, a maximum of two samples were kept for each genus for each separate clade. All sampled genera across Amaryllidaceae were retained, apart from within the American clade where sampling was reduced from 32 to 10 genera following the omission of SRA data. Protein‐coding regions of 78 plastome genes were extracted in Geneious Prime for 91 samples. Preparation of the sequence alignments and selection of models of nucleotide substitution followed the same methods and steps as detailed for the Amaryllidaceae maximum likelihood and Bayesian inference phylogenies.

At present, two fossils have been identified as potentially belonging to Amaryllidaceae: a leaf fossil (Paleocene; 58 mya; Colombia; Wing et al., 2009) and a fossil inflorescence (early Eocene; 49.42 mya; USA; Pigg et al., 2018). The leaf fossil is an undetermined monocotyledon, with an affinity to Amaryllidaceae (Wing et al., 2009), due to shared venation patterns of the fossil and extant members of Amaryllidaceae (Simpson, 2010). However, this is not considered a defining characteristic of the family (Meerow and Snijman, 1998; APG II, 2003; Simpson, 2010). Pigg et al. (2018) described a fossil inflorescence from Washington State (USA) as *Paleoallium* (Allioideae) due to morphological similarities to *Allium* and other members of Amaryllidaceae; however, Friesen (2022) disputed an Allioideae identity. The unclear taxonomic identity of these fossils prevents an accurate phylogenetic placement for estimating the divergence of Amaryllidaceae, and thus the inclusion of these fossils could introduce potential error, affecting the estimates of divergence derived (Near and Sanderson, 2004). Multiple fossil records have been identified as belonging to the Asparagales order, in which Amaryllidaceae belongs, including *Astelia antiquuea* (Maciunas et al., 2011), *Paracordyline kerguelensis* (Conran, 1997), and *Dianellophyllum eocenicum* (Conran et al., 2003). These fossils can be placed with reasonable levels of accuracy based on taxonomic identity and phylogenetic placement following assessment by Iles et al. (2015).

We used four fossils and five secondary calibration priors from the Asparagales order to constrain the tree (Table 1). The root age was set at 116.32 mya with a uniform prior, corresponding to the estimated age of the Asparagales node in Givnish et al. (2018). Fossil priors were placed with a lognormal distribution to accommodate uncertainties of fossil age and phylogenetic placement. Secondary calibration priors were placed with a uniform distribution. We estimated the date of divergence using MrBayes version 3.2.7a (Ronquist et al., 2012) with two MCMC runs with four chains. The analysis was run for 80 million generations, with sampling conducted every 8000 generations. We used a birth‐death tree prior (Gernhard, 2008) and an uncorrelated relaxed clock (Drummond et al., 2006). Tracer version 1.7.2 (Rambaut et al., 2018) was used to assess convergence between runs. The first 25% of low posterior probability trees were discarded as burn‐in.

**Table 1:** List of fossils and secondary calibration points used in the divergence dating analysis, including mean, lower, and upper ages in millions of years ago and citation information.

Taxa/group	Placement	Calibration type	Mean age	Lower age	Upper age	Source
*Astelia antiquua*	*Astelia* (Asteliaceae)	Fossil	23.20	23.20	23.20	1, 2
*Dianellophyllum eocenicum*	Hemerocallidoideae (Asphodelaceae)	Fossil	42.90	38.00	47.80	3, 4
*Paracordyline kerguelensis*	*Cordyline* (Asparagaceae)	Fossil	24.00	22.00	26.00	5, 6
*Protoyucca shadishii*	*Yucca* (Asparagaceae)	Fossil	15.35	14.50	16.20	7, 8
Asparagales	Asparagales	Secondary	116.32	111.46	121.18	9
*Asparagus/Nolina*	*Asparagus*/*Nolina* (Asparagaceae)	Secondary	43.12	39.98	46.26	9
Core Asparagales	Core Asparagales	Secondary	52.09	49.52	54.66	9
Orchidaceae	Orchidaceae	Secondary	77.61	72.75	82.47	9
Xanthorrhoeae	Xanthorrhoeae (Asphodelaceae)	Secondary	38.90	37.33	40.47	9

### Biogeographic analysis

The biogeographic analysis was conducted using BioGeoBEARS (Matzke, 2014) as implemented in RASP version 4.2 (Yu et al., 2015, 2020). The input consensus tree and tree sets were generated from the MrBayes dated phylogenetic divergence analysis. All outgroups outside of Amaryllidaceae were removed as recommended by RASP (Yu et al., 2015, 2020). The six‐model test as described by Matzke (2014) was implemented to determine the most statistically appropriate model for our data, based on the corrected Akaike information criteron (AIC_c_).

Species distributions were based on distribution data available at Plants of the World Online (POWO, 2024). Geographic areas were determined using the World Geographical Scheme for Recording Plant Distributions (Brummitt, 2001). The biogeographic areas defined for the analysis were based on prior knowledge of biogeography, centers of diversity, and natural distributions of Amaryllidaceae taxa. A total of 15 biogeographic areas were assigned (Appendix 7). Areas outside the natural range or not represented by taxa in this analysis were excluded.

## RESULTS

### Phylogenetic relationships of Amaryllidaceae

We assembled 63 new complete plastomes in this study all of which have a quadripartite structure, ranging in length from 151,685 to 166,949 bp. Seventy to seventy‐five plastome protein‐coding genes were recovered for an additional three samples. In the phylogenetic reconstructions conducted to explore the impact of missing data, gene losses, and pseudogenes, the relationships recovered between subfamilies, tribes, and groups did not differ (Appendices S8 and S9) and had high support (97–100% BS). In the analysis including only samples with 78–75 plastome protein‐coding genes, the number of genera and taxa included was reduced to 91 samples representing all subfamilies and Amaryllidoideae tribes, 38 genera, and 80 species. Additionally, the tribe Tulbagahiae of subfamily Allioideae was not represented because both samples belonging to the tribe had <75 plastome‐protein coding genes. Therefore, to include all tribes of the family and increase taxonomic breadth, we present the phylogenetic analysis including all samples of this study (49–78 plastome protein‐coding genes).

**Figure 2:**
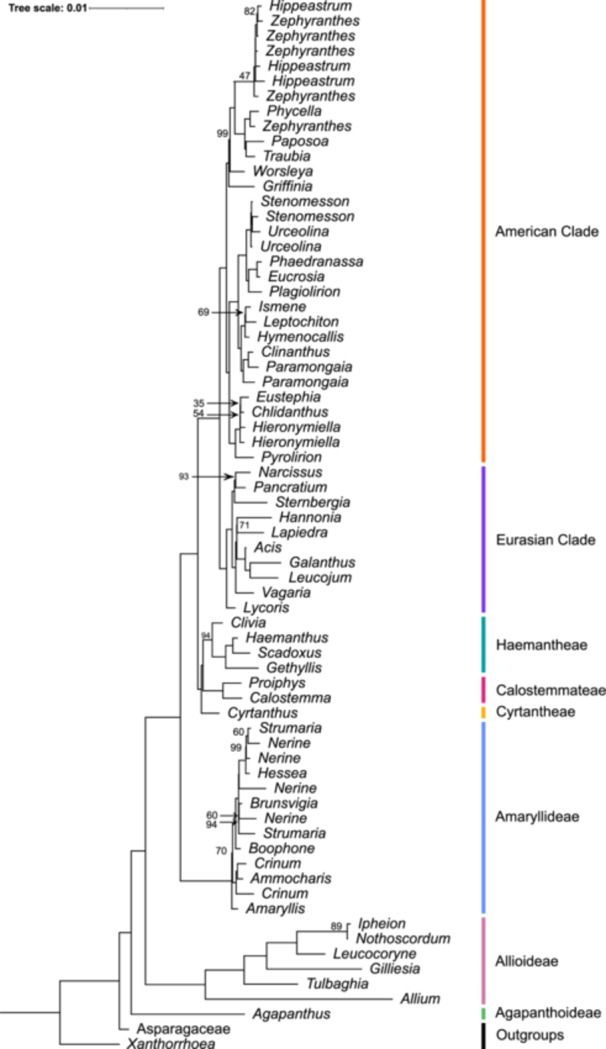
Approximated maximum likelihood phylogeny of Amaryllidaceae based on 78 plastid protein‐coding genes collapsed at genus level. Paraphyletic and polyphyletic genera were collapsed at each of their monophyletic groups. Bootstrap support values are 100% unless otherwise indicated.

The maximum likelihood and Bayesian inference analysis of the 78 plastid protein‐coding genes produced highly congruent topologies (Figure 2; Appendices S8 and S10), with minor incongruences occurring within the American clade (Appendix S11). Within tribe Eustephieae, *Eustephia darwinii*, Vargas is recovered sister to *Hieronymiella argentina* (Pax) Hunz. & S.C.Arroyo (0.77 posterior probability [PP]) in the Bayesian inference analysis, and to *Chlidanthus boliviensis* Traub & I.S.Nelson (35% bootstrap support [BS]) in the maximum likelihood analysis. The position of three *Hippeastrum* Herb. samples—*H. reginae* (L.) Herb., *H. striatum* (Lam.) H.E.Moore, and *H. vittatum* (L'Hér.) Herb.—within the Hippeastreae tribe differed between the two analyses. The Bayesian inference analysis recovered these taxa as sister to the remainder of tribe Hippeastreae (1 PP) and to *Zephyranthes bifida* (Herb.) Nic.García & Meerow and *Zephyranthes martinezii* (Ravenna) Nic.García in the maximum likelihood analysis (99% BS). The final topological incongruence between the two analyses is the relationship between members of *Griffinia* Ker Gawl. In the Bayesian inference analysis, *Griffinia alba* K.D.Preuss & Meerow is recovered sister to the remainder of the genus (1 PP), whereas *Griffinia rochae* G.M.Morel (100% BS) is sister to the remainder of the genus in the maximum likelihood analysis.

Amaryllidaceae and all three subfamilies are resolved as monophyletic (1 PP; 100% BS). Agapanthoideae is sister to Amaryllidoideae and Allioideae. Agapanthoideae consists of the monophyletic genus *Agapanthus*. High support values are recovered between all clades of the genus (1 PP; 87–100% BS). Within Allioideae all tribes are monophyletic (1 PP; 100% BS).

The American and Eurasian clades of Amaryllidoideae (*sensu* Meerow et al., 1999) are resolved as monophyletic (Table 2). All tribes are recovered as monophyletic, with the exception of Narcisseae (*Narcissus* L. and *Sternbergia* Waldst. & Kit.) and Pancratieae (*Pancratium* Dill. ex L. and *Vagaria* Herb.) in the Eurasian clade and Griffineae (*Griffinia* Ker Gawl. and *Worsleya* [Traub] Traub) in the American clade. Several genera were found to be polyphyletic within tribes Amaryllideae (*Crinum* L., *Nerine*, and *Strumaria*) and the American clade (*Hieronymiella* Pax, *Hippeastrum*, *Paramongaia* Velarde, *Phycella* Lindl., *Stenomesson* Herb., *Urceolina* Rchb., and *Zephyranthes* Herb.).

**Table 2:** Evolutionary relationships recovered by the maximum likelihood (ML) and Bayesian inference (BI) phylogenetic analyses for recognized suprageneric Amaryllidoideae groups. Posterior probability and bootstrap support are listed for each.

Amaryllidoideae group	Sister group	Support (BI/ML)
Amaryllideae	Remainder of subfamily	1 PP; 100% BS
Cyrtantheae	Calostemmateae and Haemantheae	1 PP; 100% BS
Calostemmateae	Haemantheae	1 PP; 94% BS
Haemantheae	Calostemmateae	1 PP; 94% BS
American clade	Eurasian clade	1 PP; 100% BS
Eurasian clade	American clade	1 PP; 100% BS

### Molecular dating analysis

**Figure 3:**
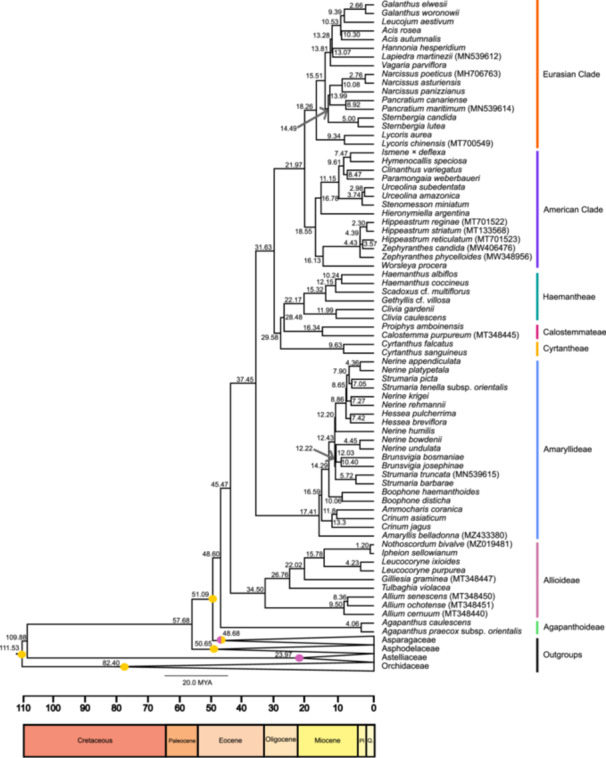
Chronogram of Amaryllidaceae inferred using MrBayes based on 78 plastome protein‐coding genes. Numbers represent median divergence ages in millions of years ago (mya). Nodes constrained by a fossil are denoted by a pink circle at the cusp of the relevant note. Nodes constrained by a secondary calibration point are denoted by a yellow circle at the cusp of the relevant node. Timings of epochs and ages listed in mya were acquired from Geological Society of America Geological Time Scale version 5.0.

The divergence analysis yielded a stem age of 51.1 mya (95% highest posterior density [HPD] = 49.5–52.6 mya) and crown age of 48.6 mya (95% HPD = 46.6–50.3 mya) in the Ypresian age of the early Eocene (56.0–47.8 mya). During the late Eocene, subfamilies Allioideae and Amaryllidoideae are estimated to have diverged 34.5 mya (95% HPD = 23.8–40.4 mya) and 37.5 mya (95% HPD = 30.5–42.3 mya), respectively. The diversification of *Agapanthus* (Agapanthoideae) began during the early Pliocene, 4.1 mya (95% HPD = 0.2–14.5 mya). Speciation of Amaryllidoideae tribes and the American and Eurasian clades started during the Miocene (Figure 3, Table 3).

**Table 3:** Estimated median and 95% highest posterior density crown and stem ages in millions of years ago for Amaryllidoideae, all subfamilies, and groups derived from the consensus dated phylogeny of this study.

Node	Crown date	Stem date
Amaryllidaceae	48.6 (46.6–50.3)	51.1 (49.5–52.6)
Amaryllidoideae	37.5 (30.5–42.3)	45.5 (42.0–48.0)
Allioideae	34.5 (23.8–40.4)	45.5 (42.0–48.0)
Agapanthus	4.1 (0.2–14.5)	48.6 (46.6–50.3)
Amaryllideae	17.4 (9.5–26.4)	37.5 (30.5–42.3)
American clade	18.6 (12.6–25.2)	22.0 (15.5–28.8)
Eurasian clade	18.3 (12.2–24.8)	22.0 (15.5–28.8)
Cyrtantheae	9.6 (3.4–18.2)	30.0 (22.3–35.8)
Calostemmateae	16.3 (9.6–23.3)	28.5 (21.3–34.9)
Haemantheae	22.2 (15.2–29.9)	28.5 (21.3–34.9)

### Biogeographic reconstruction

Of the six models tested with BioGeoBEARS (Matzke, 2014), the BAYAREALIKE model with the +j (founder event) speciation add‐on had the highest weighted AIC_c_ score (Appendix S12). Likelihood ratio tests rejected the null hypothesis indicating a statistical improvement in using the +j add‐on. The model supported 88 dispersal and five extinction events. Dispersal events occurred across the family, whereas extinction events took place within the Eurasian clade (three events) and Amaryllideae (two events).

**Figure 4:**
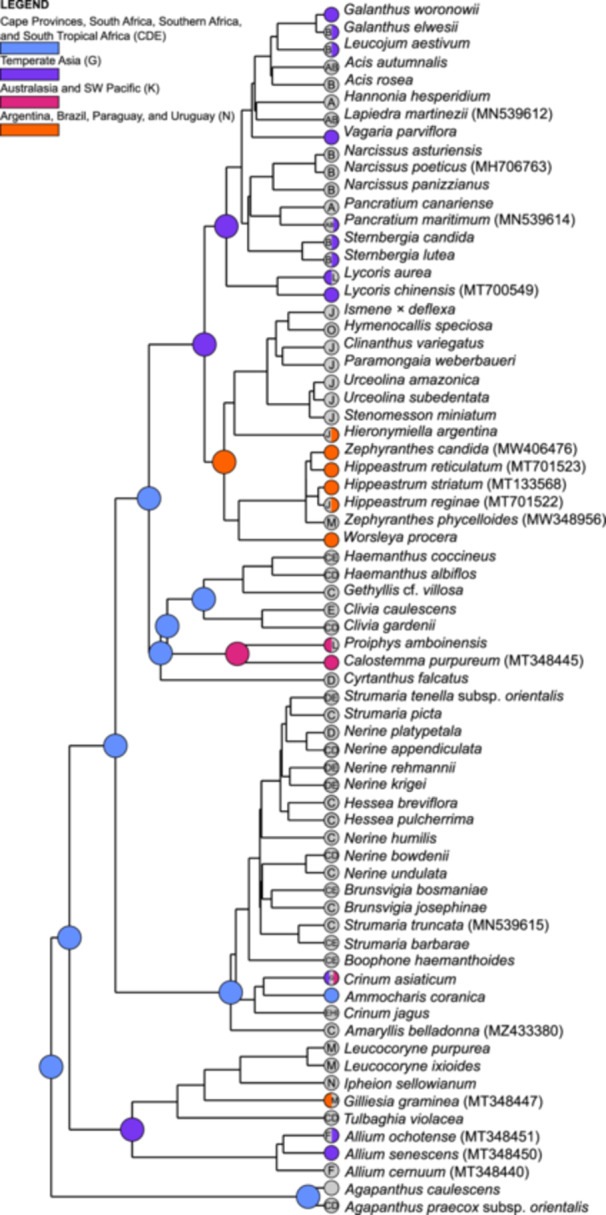
Ancestral state reconstruction of Amaryllidaceae under the BAYAREALIKE +j model using BioGeoBEARS as implemented in RASP version 4.2. Most likely ancestral area shown at internal nodes and terminal tips. A = northern Africa; B = Europe; C = Cape Provinces, South Africa; D = South Africa (excluding Cape Provinces); E = southern Africa (excluding South Africa); and south tropical Africa; F = North America; G = temperate Asia; H = west central and east tropical Africa; I = west tropical, northeast Africa, and Arabian Peninsula; J = western South America; K = Australasia and Southwest Pacific; L = tropical Asia; M = Chile; N = Argentina, Brazil, Paraguay, and Uruguay; and O = Central America and the Caribbean.

The ancestral node of Amaryllidaceae (Figure 4 and Table 4) has a southern African ancestral origin, with a likely combined origin of the Cape Provinces, South Africa, southern Africa, and south tropical Africa (state = CDE). Agapanthoideae and Amaryllidoideae are resolved to have the same ancestral origin as Amaryllidaceae (state = CDE). For Allioideae, the ancestral state could not be resolved for 57.36% of occurrences (Appendix S13), with the most likely origin determined to be the Cape Provinces and South Africa (state = CD; 16.73%).

**Table 4:** List of the most probable reconstructed ancestral origins for Amaryllidaceae, all subfamilies, and key groups based of the biogeographic reconstruction of Amaryllidaceae under the BAYAREALIKE +j model using using BioGeoBEARS as implemented in RASP version 4.2. The following regions were considered but rejected: A = northern Africa; B = Europe; F = North America; H = west central and east tropical Africa; I = west tropical, northeast Africa, and Arabian Peninsula; J = western South America; L = tropical Asia; M = Chile; and O = Central America and the Caribbean.

Node	Ancestral area probability (%)	Cape Provinces	South Africa	Southern Africa and southern tropical Africa	Temperate Asia	Australasia and Southwest Pacific	Argentina, Brazil, Paraguay, and Uruguay
Amaryllidaceae	55.43%	✓	✓	✓			
Amaryllidoideae	68.82%	✓	✓	✓			
Allioideae	16.73%	✓	✓				
*Agapanthus*	63.82%	✓	✓	✓			
Amaryllideae	87.80%	✓	✓	✓			
American clade	46.50%						✓
Eurasian clade	25.26%				✓		
Calostemmateae	58.28%					✓	
Haemantheae	72.96%	✓	✓	✓			

**Figure 5:**
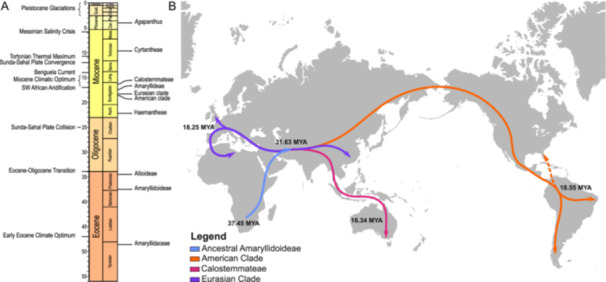
(A) Geological timeline from the Eocene to the present day. Key climatic and geological events that may have influenced Amaryllidaceae evolution are listed along with mean crown divergence ages for Amaryllidaceae, all subfamilies, and key groups. Timings of epochs and ages (in millions of years ago) were acquired from the Geological Society of America Geological Time Scale version 5.0. Initial timeline was generated using TSCreator version 8.0. (B) Illustration of predicted ancestral dispersal pathways of Amaryllidoideae groups from the Cape Provinces, South Africa, southern Africa, and south tropical Africa. Dashed lines indicate secondary colonization event.

The Amaryllidoideae tribes Amaryllideae, Haemantheae, and Cyrtantheae have an ancestral origin of southern Africa (state = CDE). Calostemmateae has an Australasian and Southwest Pacific origin, after divergence from Haemantheae following dispersal from the CDE state (Figure 5). Our analysis suggests that the Eurasian clade migrated from temperate Asia to Europe and northern Africa (15.5 mya), resulting in Asian and Mediterranean subclades, whereas taxa of the American clade migrated to southern South America (state = N) from temperate Asia.

## DISCUSSION

### Phylogenetic relationships within Amaryllidaceae

The 63 whole plastomes assembled in our study were collinear with previously published Amaryllidaceae plastomes (Zhang et al., 2020; Könyves et al., 2018, 2021), with the order of plastome protein‐coding genes consistent across the family. Thirteen of the Amaryllidoideae plastomes showed structural inverted repeat (IR) rearrangements, which has been previously reported for this subfamily by Kӧnyves et al. (2021) and Contreras‐Díaz et al. (2022), indicating further IR variability in this subfamily. Our study provides a robust plastome‐based phylogeny of Amaryllidaceae. Agapanthoideae is recovered as sister to Amaryllidoideae and Allioideae, consistent with previously published phylogenetic studies (Fay et al., 2000; Lledó et al., 2004; Pires et al., 2006; Seberg et al., 2012; Chen et al., 2013; Givnish et al., 2018; Xie et al., 2020; Dennehy et al., 2021; Kӧnyves et al., 2021; Namgung et al., 2021). We provide new insights into the evolutionary relationships of Amaryllidaceae (Figure 5A).

#### Agapanthoideae and Allioideae

The monotypic subfamily Agapanthoideae is monophyletic (Figure 2; Appendices S8 and S10). *Agapanthus* is thought to have between six and 10 species (Duncan, 2021), with considerable uncertainty over species delimitation, while the generic boundary remains firm. Within Allioideae, Allieae is resolved as sister to the remainder of the subfamily. Tulbaghieae is sister to Gilliesieae and Leucocoryneae, concurring with previous phylogenetic results using whole plastomes, two to 77 plastome protein‐coding genes, and the nuclear ribosomal cistron (Sassone and Giussani, 2018; Namgung et al., 2021; Fu et al., 2023). This is consistent with the circumscription and phylogenetic placement of tribes and genera within the subfamily (Chen et al., 2013; Sassone et al., 2014; Costa et al., 2020; Escobar et al., 2020; Namgung et al., 2021).

#### Amaryllidoideae

The relationships within Amaryllidoideae broadly concur with the findings of previous phylogenetic research (Meerow and Snijman, 2006; Chen et al., 2013), including the identification of several polyphyletic genera within the Amaryllideae tribe (*Crinum*, *Nerine*, and *Strumaria*; Meerow and Snijman, 2001; Rønsted et al., 2012), and the American clade (*Hieronymiella*, *Hippeastrum*, *Paramongaia*, *Phycella*, *Stenomesson*, *Urceolina*, and *Zephyranthes*; García et al., 2014, 2017; Meerow et al., 2000, 2020). Using implicit phylogenetic network techniques and cytonuclear discordance, the polyphyly of multiple genera within the American clade recovered in nuclear and plastome phylogenies of García et al. (2014, 2017) and Meerow et al. (2020) was attributed to reticulate evolution and ancient hybridization events. Given this evidence, and the high levels of hybridization reported to occur across the family, both in the wild and in horticulture (Marques et al., 2017; Meerow, 2023), reticulate evolution could also explain the polyphyletic relationships within the Amaryllideae tribe.

The relationships among Haemantheae, Calostemmateae, and Cyrtantheae have previously been unclear. The combined organellar and ribosomal cistron nuclear phylogeny by Rønsted et al. (2012) recovered Calostemmateae sister to Haemantheae and Cyrtantheae, with varying support (1 PP; 66% BS). In contrast, our analysis resolves Cyrtantheae sister to Haemantheae and Calostemmateae (1 PP; 96% BS), concurring with the phylogenetic analyses of Meerow and Snijman (2006) and Chen et al. (2013). Given the congruence with previous phylogenetic studies, the higher support recovered, and the inclusion of 78 plastome markers here, this is the best‐evidenced representation to date of plastid evolutionary relationships between these three tribes.

Relationships within the Eurasian clade have historically remained unresolved, including those of *Hannonia* Braun‐Blanq. & Maire, *Lapiedra* Lag., and *Vagaria* Herb. (Ito et al., 1999; Lledó et al., 2004; Meerow et al., 1999, 2006; Gage et al., 2011; Rønsted et al., 2012). Suprageneric treatments of Amaryllidoideae have included *Vagaria* within tribe Pancratieae (Traub, 1963; Dahlgren and Clifford, 1982; Meerow, 1995; Müller‐Doblies and Müller‐Doblies, 1996; Meerow and Snijman, 1998, 2001), whereas *Hannonia* and *Lapiedra* have been included in either Galantheae (Traub, 1963; Meerow, 1995; Meerow and Snijman, 1998) or Pancratieae (Müller‐Doblies and Müller‐Doblies, 1996). Our phylogenetic analysis recovered *Vagaria* as more closely related to Galantheae (*Galanthus*, *Leucojum*, *Acis*, *Hannonia*, and *Lapiedra*) than Pancratieae, which is supported by previous studies (Lledó et al., 2004; Meerow et al., 2006; Gage et al., 2011; Rønsted et al., 2012). This, therefore, confirms the current circumscription of Pancratieae as polyphyletic.

Our analysis also indicates that tribe *Narcisseae* (*Narcissus* and *Sternbergia*) is polyphyletic, with *Sternbergia* sister to *Narcissus* and *Pancratium*. This result is incongruent with previous phylogenetic studies where *Narcisseae* is monophyletic (Ito et al., 1999; Lledó et al., 2004; Meerow et al., 2006; Gage et al., 2011). The use of 78 plastome protein‐coding genes here, and multiple samples per genus, represents a step‐change in the depth of sequencing used to assess evolutionary relationships between these genera, with previous studies using one to three DNA regions. This is, therefore, an improved representation of plastid evolutionary relationships of the Eurasian clade.

Current morphological classifications used to define tribes within Amaryllidoideae do not fully align with the relationships recovered by plastome phylogenies and nuclear phylogenies. This is particularly evident for tribes of the Eurasian and American clades, but also for genera in the Amaryllideae tribe. With evidence of reticulate evolution across the American clade (García et al., 2014, 2017; Meerow et al., 2020) and hybridization in the wild and in horticulture across the family (Marques et al., 2017; Meerow, 2023), we recommend inferring phylogenetic relationships using whole plastomes and low‐copy nuclear genes prior to the revision of tribal delimitations across Amaryllioideae.

### Molecular dating analysis

The use of fossils in divergence dating studies is important in providing a minimum time for the origin of a lineage (Magallón et al., 2015; López‐Martínez et al., 2023) and helping us to constrain and validate age estimates of taxonomic groups (Iles et al., 2015; Deanna et al., 2023). The paucity of verified fossil records, particularly within herbaceous plant lineages, has limited the opportunities to estimate plant divergence times (Iles et al., 2015; Samarakoon et al., 2019; Deanna et al., 2023), including those of Amaryllidaceae and suprageneric groups (Santos‐Gally et al., 2012; Chen et al., 2013; Marques et al., 2017; Costa et al., 2020; Meerow et al., 2020; Xie et al., 2020; Namgung et al., 2021). The placement of fossils within the broader Asparagales has made it possible to estimate the divergence of this family, its subfamilies, and recognized groups with more confidence than previous publications.

Previous ages estimated for the divergence of the Amaryllidaceae family range from 87.00 to 46.77 mya (Table 5) and are derived from high‐level studies of monocotyledons (Chen et al., 2013; Givnish et al., 2018) and broader angiosperm lineages (Magallón et al., 2015; Zuntini et al., 2024). Using low‐copy nuclear genes, Zuntini et al. (2024) estimated the ages of angiosperm orders and families. They estimated a crown age of 76.6 mya in the Campanian of the late Cretaceous for Amaryllidaceae, considerably older than our Eocene age (48.6 mya; 46.6–50.3 mya). The differences in ages between the two studies could be due to differences in the following three factors. (1) *The genome used*: Plastomes, used in this study, are typically uniparentally inherited, tracing the evolution of the maternal (seed) parent (Hagemann, 2004; Wicke et al., 2011). In contrast, nuclear genomes, the low‐copy genes utilized by Zuntini et al. (2024), are biparentally inherited, reflecting the evolutionary history of both parental lineages, and can provide insights into the role of hybridization and introgression during evolution. In a family like Amaryllidaceae, in which hybridization is prevalent, the use of the plastid genome can more accurately reflect the biogeographic history of a lineage by tracking seed dispersal of the maternal lineage. Additionally, hybridization can result in discordance and rate heterogeneity between gene trees and between phylogenies constructed using different genomes (Hagemann, 2004; Linder and Rieseberg, 2004; Small et al., 2004; Wicke et al., 2011; Schneider et al., 2021). (2) *Topological differences recovered at the subfamily and family levels between the two studies*: The phylogenetic relationships recovered by Zuntini et al. (2024) were highly congruent at the tribal level; however, the relationships at the subfamily level showed incongruence to ours and to the results of previous studies (Fay et al., 2000; Lledó et al., 2004; Pires et al., 2006; Seberg et al., 2012; Chen et al., 2013; Givnish et al., 2018; Xie et al., 2020; Dennehy et al., 2021; Kӧnyves et al., 2021; Namgung et al., 2021), with Amaryllidoideae recovered as sister to Allioideae and Agapanthoideae. Additionally, the sister family of Amaryllidaceae, Asparagaceae was recovered as non‐monophyletic. (3) *The fossil priors specified*: We set the age of lower bound of *Dianellophyllum eocenicum*, the stem node of Hemerocallidoideae (Asphodelaceae), to 38.0 mya, following Iles et al. (2015); whereas Zuntini et al. (2024) set a lower bound of 41.2 mya, following the AngioCal version 1.1 database by Ramírez‐Barahona et al. (2020). This database, using dates listed by Iles et al. (2015), determines the minimum age based on either stratigraphic or radio‐isotopic ages. For *Dianellophyllum eocenicum*, the lower bound was therefore defined by the end of the Lutetian (47.8–41.2 mya) of the Eocene, explaining the differences in the age of the lower bound. To fully determine the effect of using plastome or nuclear markers to estimate divergence dates, a direct comparison should be conducted in which sampling and methods are consistent.

**Table 5:** Crown and stem ages presented to one decimal place of Amaryllidaceae, and subfamilies estimated by this and previous studies, alongside numbers of samples or genera included in analyses. The number of species sampled was not available for Wikstrӧm et al. (2001) and Janssen and Bremer (2004).

	Crown age	Stem age	Sampling
Wikström et al. (2001)			3 genera
Amaryllidoideae	33.0 mya (±4.0 mya)	55.0 mya (±5.0 mya)	2 genera
Allioideae	N/A	45.0 mya (±3.0 mya)	1 genus
Janssen and Bremer (2004)			5 genera
Amaryllidaceae	87.0 mya	91.0 mya	5 genera
Chen et al. (2013)			32 genera
Amaryllidaceae	51.2 mya (42.0–61.7 mya)	58.3 mya (50.0–67.4 mya)	41 samples
Amaryllidoideae	28.5 mya (19.2–39.4 mya)	51.2 mya (42–61.7 mya)	28 samples
Allioideae	37.0 mya (27.8–44.5 mya)	51.2 mya (42–61.7 mya)	12 samples
Agapanthoideae	N/A	51.2 mya (42–61.7 mya)	1 sample
Magallón et al. (2015)			3 genera
Amaryllidaceae	62.5 mya (49.2–76.7 mya)	N/A	9 samples
Givnish et al. (2018)			8 genera
Amaryllidaceae	46.8 mya (43.3–50.5 mya)	52.1 mya (49.4–54.7 mya)	8 samples
Amaryllidoideae	25.7 mya (21.5–30.6 mya)	46.7 mya (43.3–50.5 mya)	4 samples
Allioideae	34.7 mya (30.7–38.3 mya)	46.7 mya (43.3–50.5 mya)	3 samples
Agapanthoideae	N/A	46.7 mya (43.3–50.5 mya)	1 sample
Zuntini et al. (2024)			51 genera
Amaryllidaceae	76.6 mya	86.7 mya	53 samples
Amaryllidoideae	53.9 mya	76.7 mya	45 samples
Allioideae	51.9 mya	71.9 mya	7 samples
Agapanthoideae	N/A	71.9 mya	1 sample
This study			42 genera
Amaryllidaceae	48.6 mya (46.6–50.3 mya)	51.1 mya (49.5–52.6 mya)	72 samples
Amaryllidoideae	37.5 mya (30.5–42.3 mya)	45.5 mya (42.0–48.0 mya)	62 samples
Allioideae	34.5 mya (23.8–40.4 mya)	45.5 mya (42.0–48.0 mya)	9 samples
Agapanthoideae	4.1 mya (0.2–14.5 mya)	48.6 mya (46.6–50.3 mya)	2 samples

Our estimated crown age of Amaryllidaceae (48.6 mya; Table 3) is during the Ypresian of the early Eocene, strongly correlating with the results of Givnish et al. (2016). The Ypresian age (56.0–47.80 mya) was a period of elevated global temperatures, with reduced latitudinal temperature gradients from the tropics to the poles (Cramwinckle et al., 2018; Westerhold et al., 2020). Following the end of the Early Eocene Climate Optimum (~47 mya; Figure 5), temperatures remained elevated in comparison with modern‐day global temperatures (Westerhold et al., 2020). Hence, Amaryllidaceae diverged during the greenhouse period of the Eocene, which favored diversification, as previously suggested for other Asparagales families and subfamilies such as Agavoideae, Asparagaceae, Lomandroideae, Hemerocallidoideae, and Xanthorrhoeaceae (Chen et al., 2013; Givnish et al., 2018).

Amaryllidoideae is estimated to have diverged in the late Eocene, during which global cooling and aridification continued after the Early Eocene Climate Optimum (Westerhold et al., 2020; Hutchinson et al., 2021), as reflected by a transition from closed humid mangrove vegetation and tropical rainforests to drier and more open grasslands with seasonal aridity (Bouchenak‐Khelladi et al., 2010, 2014). Similarly, Allioideae diverged during the Eocene‐Oligocene transition, concurring with the results of Givnish et al. (2018). Similar crown ages for Allioideae are also estimated by previous divergence studies by Chen et al. (2013), Xie et al. (2020), and Namgung et al. (2021). The older divergence date by Costa et al. (2020) may reflect missing data within their alignment (20.5%) and use of a leaf fossil for calibration with an undetermined taxonomic identity (Wing et al., 2009; Meerow, 2023). The Eocene‐Oligocene transition occurred ~34 mya and lasted ~790,000 yr following an abrupt change in climate with global cooling, aridification, and the onset of the first major Antarctic glaciation (Hutchinson et al., 2021). This led to changes in floristic distributions and declines in diversity of major lineages, which were more pronounced at higher latitudes. Conversely, diversification rates increased for lineages that were better adapted to cooler and drier climates and subsequent habitat changes (Sun et al., 2014; Bitencourt et al., 2021). Amaryllidaceae is a family of geophytic plants, a trait that enables taxa to tolerate seasonal aridity (Snijman and Linder, 1996; Meerow and Snijman, 1998) and wind dispersal of seeds occurs in many genera, making them well suited to open habitats (Meerow and Snijman, 1998; Meerow et al., 2019). While it is unclear whether geophytic characteristics and mechanisms of seed dispersal evolved prior to or after the climatic changes of the late Eocene and throughout the Miocene, these characteristics have likely provided a competitive advantage for Amaryllidoideae and Allioideae lineages in light of changing climatic conditions and habitats, potentially explaining their cosmopolitan distributions in seasonally dry habitats. The diversification of these two subfamilies is therefore likely to have been initiated by changing climatic conditions and associated habitat changes, whereby species extinctions benefited taxa in both subfamilies, due to increased niche availability. Monocotyledon geophytic lineages were recovered to have higher rates of lineage diversification than non‐geophytic monocotyledons by Howard et al. (2019); this is hypothesized to be driven by shifts in climate, supporting our results. Given the cosmopolitan distribution and broad range of habitats Amaryllidaceae taxa inhabit, ancestral state reconstruction and determination of niche partitioning between bulbous and rhizomatous taxa could identify abiotic and biotic drivers of geophytic evolution and transition within and beyond the family.

All Amaryllidoideae groups recognized in our study (Table 3) diverged during the Miocene, an epoch of progressive cooling and aridity marked by periods of warmer conditions with climatic events such as the Miocene Climatic Optimum (16.74–14.5 mya) and Tortonian Thermal Maximum (10.75 mya) (Westerhold et al., 2020; Boettner et al., 2021). The African tribes—Amaryllideae, Haemantheae, and Cyrtantheae—occur predominantly in Africa, with centers of diversity in South Africa (Meerow et al., 1999). *Crinum* (Amaryllideae) and *Scadoxus* (Haemantheae) are exceptions, with *Crinum* occurring in tropical and subtropical regions globally (Meerow et al., 2003), and *Scadoxus* occurring from tropical Africa to the Arabian Peninsula (Collenette, 1985; Wood et al., 1997; Duncan et al., 2020). During the Miocene, tropical and subtropical areas including the Cape Floristic Region provided climatic refugia from aridification for many plant lineages (Goldblatt and Manning, 2002; Braun et al., 2023), while southern Africa provided refugia during the repeated glacial periods of the Quaternary (2.58 mya to present; Braun et al., 2023). The restricted distributions of Amaryllideae (except *Crinum*), Haemantheae, and Cyrtantheae therefore may reflect range contraction during cooler periods of the Miocene and glacial periods of the Quaternary, with their current distributions in consequence of more recent palaeoclimates (Meerow et al., 1999). The climatic instability of the Miocene, alongside cooling and glacial periods throughout the epoch (Meerow et al., 1999; Cramwinckle et al., 2018; Westerhold et al., 2020), likely resulted in climate‐induced divergence between Calostemmateae and Haemantheae, as well as between the American and Eurasian tribes.

### Biogeographic history of Amaryllidaceae

Amaryllidaceae and subfamily Amaryllioideae originated in southern Africa and south tropical Africa, supporting previous biogeographic analyses for subfamilies Amaryllidoideae (Ito et al., 1999; Meerow et al., 1999) and Allioideae (Costa et al., 2020; Namgung et al., 2021). This reflects the current‐day center of diversity of the family, with members belonging to each subfamily native to the proposed ancestral area (Meerow, 2023). Additionally, Amaryllidoideae has centers of diversity in South Africa and southern Africa (Ito et al., 1999; Meerow et al., 2020). We could not estimate the ancestral origin of Allioideae with high certainty. This is likely due to the limited sampling, particularly for *Allium*, which consists of ~1000 species (Zhang et al., 2024), combined with the intercontinental disjunct distributions of Allioideae. A previous biogeographic study of Allioideae by Namgung et al. (2021) indicated an African origin complementing our results, which suggested a potential ancestral origin of South Africa. We therefore corroborate an African origin for Allioideae, with an origin in southern Africa as the most probable biogeographic history for this subfamily based on current evidence. We propose that global cooling and increasing aridity throughout the Eocene enabled range expansion and lineage diversification of Amaryllidoideae and Allioideae, with the Arabian Peninsula a key biogeographic route in explaining the cosmopolitan distribution of these subfamilies.

The Amaryllideae tribe is estimated to have diverged in southern Africa and south tropical Africa during the Miocene. All genera within the tribe are endemic to the continent, with several genera endemic to South Africa (Meerow et al., 1999), with the exception of *Crinum* (Meerow et al., 2003). The wide distribution of *Crinum* (Africa, Australia, North America, South America, and across much of Asia) can be explained by long‐distance dispersal across oceanic routes (Meerow et al., 2003), with seeds of the genus well suited for oceanic dispersal and biotic dispersal not otherwise recorded for Amaryllideae (Meerow and Snijman, 1998).

Calostemmateae, which is distributed from Southeast Asia to Australia and Melanesia (Meerow et al., 1999; Meerow, 2023), is sister to the African and southeast Arabian tribe Haemantheae (Collenette, 1985; Wood et al., 1997; Duncan et al., 2020). The distribution of Calostemmateae was previously explained by migration to Australia from Africa prior to the separation of these landmasses (Ito et al., 1999; Meerow et al., 1999). The East Gondwana breakup began ~140 mya, resulting in the separation of Africa, Antarctica, Australia, India, and Madagascar (Seton et al., 2012). This is significantly older than both our estimate (48.6 mya) and previous estimates (87–46.8 mya; Janssen and Bremer, 2004; Chen et al., 2013; Magallón et al., 2015; Givnish et al., 2018; Zuntini et al., 2024) proposed for the divergence of Amaryllidaceae. The seeds of *Calostemma*, though buoyant, are intolerant to prolonged submersion in seawater (Clark and Parsons, 1994), ruling out dispersal to Australia and Southeast Asia by oceanic currents. We therefore suggest an alternative biogeographic route for this tribe in which Calostemmateae migrated through temperate Asia via the Arabian Peninsula, into Southeast Asia, and then followed the route of the Sunda‐Sahal floristic exchange before diversifying in Melanesia and Australia.

Following dispersal to temperate Asia via the Arabian Peninsula, the disjunct distributions of the American and Eurasian clades of Amaryllioideae can be explained by cooling and aridification of Central Asia due to the progressive uplift of the Qinghai‐Tibet Plateau (Royden et al., 2008), which resulted in climate‐initiated speciation and divergence. This has also been proposed to explain the disjunct distributions between *Theligonum* and *Kelloggia* (Rubiaceae; Deng et al., 2017), and within *Rhodiola* (Crassulaceae; Zhang et al., 2014), as well as the divergence and diversification of *Polygonatum* (Asparagaceae; Wang et al., 2016) and *Hedysarum* (Fabaceae; Juramurodov et al., 2023) in the Miocene. The American clade followed the Beringian migration pathway, dispersing to North America and then South America. The cooling of the early Miocene (Seton et al., 2012; Cramwinckle et al., 2018; Westerhold et al., 2020; Denk et al., 2023) and subsequent glacial periods (Meerow et al., 1999) is likely to have further restricted the American clade to more subtropical and tropical latitudes, explaining the center of diversity of American Amaryllids in South America. The Eurasian clade diversified within temperate Asia and across Europe, occupying predominantly temperate and semiarid regions, explaining present‐day distributions. The migration of taxa in northern Africa is likely to have occurred during multiple climatic and geological events such as the Messinian salinity crisis (5.97–5.33 mya) and the Pleistocene Glaciations (from 2.5 mya to 11,700 yr ago), which facilitated movement between the Iberian Peninsula and northern Africa along the Strait of Gibraltar (Krijgsman et al., 2018). This could explain the presence of genera such as *Acis*, *Lapiedra*, and *Narcissus* on both sides of the Strait of Gibraltar. The distribution of *Pancratium* across Africa, Europe, and Asia can be explained by secondary dispersal events, with *Pancratium* seeds resistant to submergence in seawater (De Castro et al., 2020).

## CONCLUSIONS

Our phylogenetic analysis provides the clearest insight into the plastome evolutionary relationships of Amaryllidaceae. Amaryllidaceae and all subfamilies are recovered as monophyletic; however, current morphological classifications show incongruence to the relationships recovered by plastome phylogenies of several Eurasian and American tribes, and genera of Amaryllideae and within the American clade. Prior to revising tribal delimitations of Amaryllidoideae, we recommend assessing cytonuclear discordance between plastome and nuclear phylogenies due to the extent of reticulate evolution already inferred for Amaryllidaceae taxa.

Given the cosmopolitan distribution and Eocene origin of Amaryllidaceae, understanding dispersal mechanisms and routes of the family also provides us with insights into the biogeographic histories of other plant groups that present out‐of‐Africa distributions and that diverged following the separation of Gondwana. Our analysis indicates that continental migration across the Arabian Peninsula to temperate Asia is important in explaining the cosmopolitan distribution of Allioideae and Amaryllidoideae. Other key biogeographic corridors and gateways include the Bering land bridge, central Asian gateway, and Sunda‐Sahal floristic exchange. The dated Amaryllidaceae phylogeny and biogeographic analysis presented here provide a backbone for understanding the biogeographic history of suprageneric groups and genera within the family, enabling a new understanding of the drivers and processes that have shaped their evolutionary histories.

## AUTHOR CONTRIBUTIONS

Z.D.C., A.C., J.D., K.K., and C.Y. conceived the study. Z.D.C. and K.K. conducted lab work prior to sequencing and assembled plastome data. Z.D.C. analyzed the data and wrote the first version of the manuscript. All authors read, edited, and approved the manuscript.

## Supporting information


**Appendix S1.** Amaryllidoideae tribes groups used in this study.
**Appendix S2.** List of new plastomes constructed for this study, including voucher information, GenBank accessions, and length of the whole plastome.
**Appendix S3.** List of taxa acquired from previous publications, including GenBank or SRA accessions and citation information.
**Appendix S4.** Taxa used as starting seeds for GetOrganelle assemblies of the SRA data used.
**Appendix S5.** Asparagales taxa used to place fossils and secondary dates for the divergence analysis, with source and collection vouchers.
**Appendix S6.** Taxa included in the wider Asparagales dated phylogeny.
**Appendix S7.** Biogeographic areas assigned using the World Geographical Scheme for Recording Plant Distributions.
**Appendix S8.** Maximum likelihood phylogeny of Amaryllidaceae based on 78 plastid protein‐coding genes.
**Appendix S9.** Maximum likelihood consensus phylogeny of Amaryllidaceae based on 75–78 plastid protein‐coding genes.
**Appendix S10.** Bayesian inference consensus phylogeny of Amaryllidaceae based on 78 plastid protein‐coding genes.
**Appendix S11.** Tanglegram between plastome maximum likelihood and Bayesian inference phylogenies of the American clade showing incongruence between the two analyses.
**Appendix S12.** AIC_c_ statistic scores for BioGeoBEARS biogeographic analysis conducted using RASP version 4.2.
**Appendix S13.** List of the four most probable reconstructed ancestral origins for Amaryllidaceae, all subfamilies, and key groups.

## Data Availability

Newly generated raw sequence reads are deposited in the Sequence Read Archive database (bioproject no. PRJNA1208551: Appendix S2). Newly constructed whole plastomes are deposited in GenBank. GenBank accession numbers are listed in Appendix S2. Sequence alignments and phylogenetic tree files are deposited in https://www.qiagen.com/us/resources/resourcedetail?id=6b9bcd96-d7d4-48a1-9838-58dbfb0e57d0&lang=en.
