# Supplementary material for: The origin and diversification of Amaryllidaceae: A phylogenetic and biogeographic analysis
Source: Am J Bot. 2025 Sep 11;112(9):e70092. doi: 10.1002/ajb2.70092 (PMC12464465; doi:10.1002/ajb2.70092)
Supplement: Supplementary file 1 — Appendix S1. Amaryllidoideae tribes groups used in this study. Appendix S2. List of new plastomes constructed for this study, including voucher information, GenBank accessions, and length of the whole plastome. Appendix S3. List of taxa acquired from previous publications, including GenBank or SRA accessions and citation information. Appendix S4. Taxa used as starting seeds for GetOrganelle assemblies of the SRA data used. Appendix S5. Asparagales taxa used to place fossils and secondary dates for the divergence analysis, with source and collection vouchers. Appendix S6. Taxa included in the wider Asparagales dated phylogeny. Appendix S7. Biogeographic areas assigned using the World Geographical Scheme for Recording Plant Distributions. Appendix S8. Maximum likelihood phylogeny of Amaryllidaceae based on 78 plastid protein‐coding genes. Appendix S9. Maximum likelihood consensus phylogeny of Amaryllidaceae based on 75–78 plastid protein‐coding genes. Appendix S10. Bayesian inference consensus phylogeny of Amaryllidaceae based on 78 plastid protein‐coding genes. Appendix S11. Tanglegram between plastome maximum likelihood and Bayesian inference phylogenies of the American clade showing incongruence between the two analyses. Appendix S12. AICc statistic scores for BioGeoBEARS biogeographic analysis conducted using RASP version 4.2. Appendix S13. List of the four most probable reconstructed ancestral origins for Amaryllidaceae, all subfamilies, and key groups. [file AJB2-112-e70092-s001.zip › Appendix_S2.docx]

Appendix S2 – List of new plastomes constructed for this study, including voucher information, GenBank accessions and length of the whole plastome. Material acquired from Bristol = University of Bristol Botanic Garden, De Hortus = Hortus Botanicus, JC = Jamie Compton, JD = John David, K = Royal Botanic Gardens, Kew, KK = Kálmán Könyves, Meise = Plantentuin Meise, PC = Paul Cumbeton, RNG = University of Reading, RBGE = Royal Botanic Garden Edinburgh, and WSY = Royal Horticultural Society Wisley.

| **Taxon** | **GenBank Accession** | **SRA Accession** | **Origin** | **Voucher** |
| --- | --- | --- | --- | --- |
| *Acis autumnalis* | PP853182 | SRR31993683 | JD | WSY0177153 |
| *Acis rosea* | PP853187 | SRR31993638 | JD | No voucher |
| *Agapanthus caulescens* | PP853183 | SRR31993682 | RBGE (19653552A) | WSY0177101 |
| *Agapanthus praecox*subsp*. orientalis* | PP853184 | SRR31993671 | RBGE (19912195B) | WSY0177102 |
| *Amaryllis belladonna* | PP853185 | SRR31993660 | WSY | WSY0177103 |
| *Ammocharis coranica* | PP853186 | SRR31993649 | Bristol | WSY0177104 |
| *Boophone disticha* | PP853190 | SRR31993619 | WSY | WSY0177105 |
| *Boophone haemanthoides* | PP853189 | SRR31993620 | WSY | WSY0177106 |
| *Brunsvigia bosmaniae* | PP853191 | SRR31993618 | WSY | WSY0177107 |
| *Brunsvigia gregaria* | PP853192 | SRR31993681 | WSY | WSY0177108 |
| *Brunsvigia josephinae* | PP853193 | SRR31993680 | WSY | WSY0177109 |
| *Clinanthus variegatus* | PP853199 | SRR31993674 | JD | WSY0177110 |
| *Clivia caulescens* | PP853196 | SRR31993677 | RBGE | WSY0177111 |
| *Clivia gardenii* | PP853197 | SRR31993676 | WSY | WSY0177112 |
| *Crinum asiaticum* | PP853198 | SRR31993675 | RBGE | WSY0177113 |
| *Crinum jagus* | PP853195 | SRR31993678 | RBGE | WSY0177114 |
| *Cyrtanthus falcatus* | PP853200 | SRR31993673 | WSY | WSY0177115 |
| *Cyrtanthus sanguineus* | PQ684178 – PQ684254 | SRR31993621 | WSY | WSY0177116 |
| *Galanthus elwesii* | PP853202 | SRR31993670 | WSY | WSY0177117 |
| *Galanthus reginae*-*olgae* | PP853203 | SRR31993669 | WSY | WSY0177118 |
| *Galanthus woronowii* | PP853204 | SRR31993668 | WSY | WSY0177119 |
| *Gethyllis villosa* | PP853205 | SRR31993667 | WSY | WSY0177120 |
| *Haemanthus albiflos* | PP853209 | SRR31993663 | WSY | WSY0177121 |
| *Haemanthus coccineus* | PP853206 | SRR31993666 | WSY | WSY0177122 |
| *Haemanthus humilis* | PP853208 | SRR31993664 | JC | WSY0177123 |
| *Hannonia hesperidium* | PP853207 | SRR31993665 | K (JUTU 14314) | K000365499 |
| *Hessea breviflora* | PP853210 | SRR31993662 | De Hortus (20110213) | WSY0156590 |
| *Hessea matthewsii* | PP853211 | SRR31993661 | De Hortus (20110218) | No voucher |
| *Hessea pulcherrima* | PP853212 | SRR31993659 | PC | WSY0177152 |
| *Hieronymiella argentina* | PP853213 | SRR31993658 | WSY | WSY0177124, WSY0177125 |
| *Hymenocallis speciosa* | PP853214 | SRR31993657 | RBGE (19592060A) | WSY0156591 |
| *Ipheion sellowianum* | PP853216 | SRR31993655 | WSY | WSY0177126 |
| *Ismene* × *deflexa* | PP853215 | SRR31993656 | KK | WSY0177127 |
| *Leucocoryne ixioides* | PP853218 | SRR31993653 | WSY | WSY0177128 |
| *Leucocoryne purpurea* | PP853219 | SRR31993652 | WSY | WSY0177129 |
| *Leucojum aestivum* | PP853217 | SRR31993654 | WSY | WSY0177130 |
| *Lycoris aurea* | PP853220 | SRR31993651 | RBGE | E00421356 |
| *Narcissus asturiensis* | PP853188 | SRR31993627 | RNG (PMA-1) |  |
| *Narcissus fernandesii* | PP853221 | SRR31993650 | RBGE (19892050A) | WSY0156592 |
| *Narcissus panizzianus* | PP853229 | SRR31993641 | JD | No voucher |
| *Nerine appendiculata* | PP853222 | SRR31993648 | GRA | J David NAASE2 |
| *Nerine bowdenii* | PP853223 | SRR31993647 | GRA | J David NAASE1 |
| *Nerine humilis* | PP853228 | SRR31993642 | WSY | WSY0177133 |
| *Nerine krigei* | PP853224 | SRR31993646 | WSY | WSY0177134 |
| *Nerine masoniorum* | PP853225 | SRR31993645 | WSY | WSY0177135 |
| *Nerine platypetala* | PP853226 | SRR31993644 | WSY | WSY0177136 |
| *Nerine rehmannii* | PP853227 | SRR31993643 | WSY | WSY0177137 |
| *Nerine undulata* | PQ684107 – PQ684177 | SRR31993623 | GRA | J David NAASE7 |
| *Pancratium canariense* | PP853230 | SRR31993640 | Meise (19792812) | BR0000025668421V |
| *Paramongaia weberbaueri* | PP853231 | SRR31993639 | WSY | WSY0177139, WSY0177140 |
| *Proiphys amboinensis* | PP853232 | SRR31993637 | Meise (19700627) | BR0000022719249 |
| *Scadoxus multiflorus* | PP853233 | SRR31993636 | KK | WSY0177141 |
| *Stenomesson miniatum* | PP853236 | SRR31993633 | WSY | WSY0177142 |
| *Sternbergia candida* | PP853234 | SRR31993635 | WSY | WSY0177143 |
| *Sternbergia lutea* | PP853235 | SRR31993634 | WSY | WSY0177144 |
| *Strumaria barbarae* | PP853237 | SRR31993632 | De Hortus (20110212) | WSY0156593 |
| *Strumaria discifera* | PP853238 | SRR31993631 | WSY | WSY0177145 |
| *Strumaria gemmata* | PP853239 | SRR31993630 | De Hortus (20110217) | WSY0156594 |
| *Strumaria phonolithica* | PP853240 | SRR31993629 | WSY | WSY0177146 |
| *Strumaria picta* | PP853241 | SRR31993628 | De Hortus (20110221) | WSY0156595 |
| *Strumaria tenella* subsp*. orientalis* | PP853242 | SRR31993626 | WSY | WSY0177147 |
| *Tulbaghia violacea* | PQ684037 – PQ684106 | SRR31993622 | WSY | WSY0177150 |
| *Urceolina amazonica* | PP853201 | SRR31993672 | WSY | WSY0177148, WSY0177149 |
| *Urceolina subedentata* | PP853194 | SRR31993679 | Meise (19670106) | BR0000025668414V |
| *Vagaria parviflora* | PP853243 | SRR31993625 | JD | WSY0177151 |
| *Worsleya procera* | PP853244 | SRR31993624 | WSY | WSY0177131, WSY0177132 |
