# Supplementary material for: The origin and diversification of Amaryllidaceae: A phylogenetic and biogeographic analysis
Source: Am J Bot. 2025 Sep 11;112(9):e70092. doi: 10.1002/ajb2.70092 (PMC12464465; doi:10.1002/ajb2.70092)
Supplement: Supplementary file 1 — Appendix S1. Amaryllidoideae tribes groups used in this study. Appendix S2. List of new plastomes constructed for this study, including voucher information, GenBank accessions, and length of the whole plastome. Appendix S3. List of taxa acquired from previous publications, including GenBank or SRA accessions and citation information. Appendix S4. Taxa used as starting seeds for GetOrganelle assemblies of the SRA data used. Appendix S5. Asparagales taxa used to place fossils and secondary dates for the divergence analysis, with source and collection vouchers. Appendix S6. Taxa included in the wider Asparagales dated phylogeny. Appendix S7. Biogeographic areas assigned using the World Geographical Scheme for Recording Plant Distributions. Appendix S8. Maximum likelihood phylogeny of Amaryllidaceae based on 78 plastid protein‐coding genes. Appendix S9. Maximum likelihood consensus phylogeny of Amaryllidaceae based on 75–78 plastid protein‐coding genes. Appendix S10. Bayesian inference consensus phylogeny of Amaryllidaceae based on 78 plastid protein‐coding genes. Appendix S11. Tanglegram between plastome maximum likelihood and Bayesian inference phylogenies of the American clade showing incongruence between the two analyses. Appendix S12. AICc statistic scores for BioGeoBEARS biogeographic analysis conducted using RASP version 4.2. Appendix S13. List of the four most probable reconstructed ancestral origins for Amaryllidaceae, all subfamilies, and key groups. [file AJB2-112-e70092-s001.zip › Appendix_S3.docx]

**Appendix S3** – List of taxa acquired from previous publications, including GenBank or SRA accessions and citation information. Herbarium codes: ANUB =Anhui Normal University, B = ZE Botanischer Garten und Botanisches Museum, Freie Universität Berlin, CONC = Herbario Universidad de Concepción, CUVC =Universidad del Valle, CWU =V. N. Karazin National University, E = Royal Botanic Garden Edinburgh, F = Field Museum of Natural History, FLAS = Florida Museum of Natural History, FSU = Florida State University, FTG = Fairchild Tropical Botanic Garden, GAAS = Guangdong Academy of Agricultural Sciences, GCU = Gachon University, HAO = Universidad Privada Antenor Orrego, HEAC = Henan Agricultural University, HMJAU = Jilin Agricultural University, HZU = Zhejiang University, JBN = Fundacion Jardin Botanico Nacional, JGSU = Jinggangshan University, K = Royal Botanic Gardens Kew, KH = Korea National Arboretum, KRIB = Korea Research Institute of Bioscience and Biotechnology, LNAF = Liaoning Academy of Foresty, MO = Missouri Botanical Garden, NA = United States National Arboretum, USDA-ARS, NAS = Institute of Botany, Jiangsu Province and Chinese Academy of Sciences, NF = Nanjing Forestry University, OSBU = Universität Osnabrück, SCHG = Garden Route Botanical Garden, SEL = Marie Selby Botanical Gardens, SGO = Museo Nacional de Historia Natural, SZG = Fairy Lake Botanical Garden, UEC = Universidade Estadual de Campinas, UOA = Universidad de Atacama, WIS = University of Wisconsin, WSY = Royal Horticultural Society Wisley, XYTC = Xinyang Agriculture and Forestry University, ZIT = Zhangzhou Institute of Technology.

| **Taxon** | **Accession** | **Reference** | **Voucher** |
| --- | --- | --- | --- |
| *Acis autumnalis* var. *oporantha* | MN539611 | Kӧnyves et al. (2021) | WSY0153095 (WSY) |
| *Agapanthus coddii* | MT348439 | Namgung et al. (2021) | Chase 20081397 (K) |
| *Agapanthus praecox* | MW829770 | Dong et al. (2021) | YJH01 (XYTC) |
| *Allium cernuum* | MT348440 | Namgung et al. (2021) | Namgung 20170811 (WIS) |
| *Allium karataviense* | MT348442 | Namgung et al. (2021) | Choi 11052015-8 (CWU) |
| *Allium koreanum* | MT348449 | Namgung et al. (2021) | Choi 2015622 (CWU) |
| *Allium ochotense* | MT348451 | Namgung et al. (2021) | Kim 04-1421 (GCU) |
| *Allium senescens* | MT348450 | Namgung et al. (2021) | Namgung 20170811 (GCU) |
| *Amaryllis belladonna* | MZ433380 | Dennehy et al. (2021) | WSY0150055 (WSY) |
| *Calostemma purpureum* | MT348445 | Namgung et al. (2021) | Namgung 20180430 (GCU) |
| *Chlidanthus boliviensis* | SRR11927367 | Meerow et al. (2020) | Doran-44 (MO) |
| *Clinanthus humilis* | SRR11927345 | Meerow et al. (2020) | Herklotts-s.n. (K) |
| *Clinanthus incarnatus* | SRR11927312 | Meerow et al. (2020) | Munn-136 (K) |
| *Clivia caulescens* | MW660366 | Wu et al. (2021) | YJJZL04 (LNAF) |
| *Clivia gardenii* | MW561117 | Wu et al. (2021) | HYJZL02 (LNAF) |
| *Clivia miniata* | MN857162 | Wang et al. (2020) | NEF120190802WW1 (HMJAU) |
| *Clivia robusta* | MW660367 | Zhao et al. (2022) | ZZJZL03 (LNAF) |
| *Cordyline indivisa* | KX822776 | N/A | 20171291(K) |
| *Crinum asiaticum* var. *pedunculatum* | MT348448 | Namgung et al. (2021) | Park 140806 (GCU) |
| *Cyrtanthus mackenii* | MT348446 | Namgung et al. (2021) | Namgung 20171110 (KH) |
| *Eucrosia bicolor* | SRR11927376 | Meerow et al. (2020) | Plowman 54512 (F) |
| *Eucrosia mirabilis* | SRR11927374 | Meerow et al. (2020) | Meerow-3612 (NA) |
| *Eucrosia stricklandii* | SRR11927373 | Meerow et al. (2020) | Plowman 14356A (SEL) |
| *Eustephia darwinii* | SRR11927372 | Meerow et al. (2020) | Meerow-2436 (FTG) |
| *Gilliesia graminea* | MT348447 | Namgung et al. (2021) | Chase 450 (K) |
| *Griffinia alba* | SRR11927371 | Meerow et al. (2020) | Campos-Rocha 1478 (UEC) |
| *Griffinia hyacinthina* | SRR3183890 | Garcia et al. (2014) | Meerow 2106 (FTG, FLAS) |
| *Griffinia nocturna* | SRR3185235 | Garcia et al. (2014) | Dutilh sn (UEC) |
| *Griffinia rochae* | SRR3189192 | Garcia et al. (2014) | Dutilh sn (UEC) |
| *Hieronymiella argentina* | SRR11927369 | Meerow et al. (2020) | Chase-1901 (K) |
| *Hippeastrum brasilianum* | SRR3183895 | Garcia et al. (2014) | Meerow 2405 (FTG) |
| *Hippeastrum reginae* | MT701522 | Liu et al. (2022a) | GAAS |
| *Hippeastrum reticulatum* | MT701523 | Liu et al. (2022a) | GAAS |
| *Hippeastrum reticulatum* | SRR3185380 | Garcia et al. (2014) | Meerow 2407 (FTG) |
| *Hippeastrum striatum* | MT133568 | Huang (2020) | ZITP2019830 (ZIT) |
| *Hippeastrum vittatum* | MT762362 | Li et al. (2020a) | SZG00057319 (SZG) |
| *Hymenocallis glauca* | SRR11927348 | Meerow et al. (2020) | Meerow-2433 (FTG) |
| *Hymenocallis godfreyi* | SRR11927347 | Meerow et al. (2020) | Godfrey-83721 (FSU) |
| *Hymenocallis leavenworthii* | SRR11927339 | Meerow et al. (2020) | Leavenworth-651 (F) |
| *Ismene amancaes* | SRR11927320 | Meerow et al. (2020) | Meerow-2452 (FTG) |
| *Ismene vargasii* | SRR11927319 | Meerow et al. (2020) | Meerow-2308 (FTG) |
| *Lapiedra martinezii* | MN539612 | Kӧnyves et al. (2021) | WSY0153096 (WSY) |
| *Leptochiton quitoensis* | SRR11927318 | Meerow et al. (2020) | Plowman-5411 (F) |
| *Leucojum aestivum* | MH422130 | Li et al. (2018) | L20180318 (HZU) |
| *Lycoris anhuiensis* | MT700550 | Zhang et al. (2020) | NAS00585494 (NAS) |
| *Lycoris aurea* | MN831471 | Zhang et al. (2020) | NAS00585496 (NAS) |
| *Lycoris chinensis* | MK353217 | Liu et al. (2022b) | LC08002 (ANUB) |
| *Lycoris chinensis* | MT700549 | Zhang et al. (2020) | NAS00591936 (NAS) |
| *Lycoris longituba* | MN096601 | Zhang et al. (2019a) | NAS00585500 (NAS) |
| *Lycoris radiata* | MN158120 | Zhang et al. (2019b) | Lra-3 (NAS) |
| *Lycoris sanguinea* | MK353220 | Liu et al. (2022b) | LK1005 (ANUB) |
| *Lycoris sprengeri* | MN158986 | Zhang et al. (2019c) | NAS00585503 (NAS) |
| *Lycoris squamigera* | MH118290 | Jin et al. (2018) | 37°05'41.6″N 127°24'23.4″E; Photo (KRIB) |
| *Narcissus poeticus* | MH706763 | Kӧnyves et al. (2018) | WSY0108940 (WSY) |
| *Narcissus tazetta* subsp. *chinensis* | MN432153 | Li et al. (2020b) | NF000015 (NF) |
| *Nerine sarniensis* | MN539613 | Kӧnyves et al. (2021) | WSY0108940 (WSY) |
| *Nothoscordum bivalve* | MZ019481 | Scobeyeva et al. (2021) | 01-17-0026-10 (OSBU) |
| *Nothoscordum bonariense* | MT348455 | Namgung et al. (2021) | Brownless 19811006 (E) |
| *Pancratium maritimum* | MN539614 | Kӧnyves et al. (2021) | WSY0153098 (WSY) |
| *Pancratium zeylanicum* | SRR11927315 | Meerow et al. (2020) | Meerow-3604 (NA) |
| *Paramongaia milagroantha* | SRR11927314 | Meerow et al. (2020) | Leiva & Leiva-5795 (HAO) |
| *Phaedranassa carmiolii* | SRR11927309 | Meerow et al. (2020) | Hammel-26660 (MO) |
| *Phaedranassa cinerea* | SRR11927308 | Meerow et al. (2020) | Meerow & Meerow-1045 (FTG) |
| *Phaedranassa dubia* | SRR11927306 | Meerow et al. (2020) | Meerow-1129 (FTG) |
| *Phycella arzae* | SRR3184080 | Garcia et al. (2014) | Meerow 3100 (FTG) |
| *Phycella chilensis* | SRR3185393 | Garcia et al. (2014) | Escobar 35 (CONC) |
| *Phycella cyrtanthoides* | SRR3189430 | Garcia et al. (2014) | N. García 4163 (FLAS; CONC) |
| *Phycella davidii* | SRR3189431 | Garcia et al. (2014) | N. Garcia 3031/G40 (FLAS, CONC) |
| *Phycella maulensis* | SRR3185225 | Garcia et al. (2014) | N. García 4384/G43 (SGO) |
| *Phycella ornata* | SRR3185390 | Garcia et al. (2014) | Fernández 68 (JBN) |
| *Paposa laeta* | SRR3185392 | Garcia et al. (2014) | N. Garcíá 1022 (FLAS) |
| *Phycella scarlatina* | SRR3189430 | Garcia et al. (2014) | N. García 862/G14 (CONC) |
| *Plagiolirion horsmannii* | SRR11927305 | Meerow et al. (2020) | Silverstone-Sopkin-6520 (NA) |
| *Pyrolirion tubiflorum* | SRR11927403 | Meerow et al. (2020) | Meerow-3109 (NA) |
| *Stenomesson ecuadorense* | SRR11927397 | Meerow et al. (2020) | Jost-7949 (NA) |
| *Stenomesson flavum* | SRR11927396 | Meerow et al. (2020) | Meerow-2430 (FTG) |
| *Stenomesson korsakoffii* | SRR11927390 | Meerow et al. (2020) | Meerow-1096 (FLAS) |
| *Stenomesson leucanthum* | SRR11927395 | Meerow et al. (2020) | Meerow-2522 (FTG) |
| *Strumaria truncata* | MN539615 | Kӧnyves et al. (2021) | WSY0153099 (WSY) |
| *Traubia modesta* | SRR3184145 | Garcia et al. (2014) | N. García 4357/G88 (FLAS) |
| *Tulbaghia violacea* | MT323239 | Namgung et al. (2021) | Namgung 20181023 (SCHG) |
| *Urceolina formosa* | SRR11927381 | Meerow et al. (2020) | Whitten et al.-95020 (FLAS) |
| *Urceolina subedentata* | SRR11927389 | Meerow et al. (2020) | Meerow-1109 (FLAS) |
| *Urceolina tenera* | SRR11927378 | Meerow et al. (2020) | Cano-Palacios-s.n. (CUVC) |
| *Worsleya procera* | SRR11927391 | Meerow et al. (2020) | Meerow-2411 (FTG) |
| *Zephyranthes advena* | SRR3184124 | Garcia et al. (2014) | N. García 2964 (FLAS, CONC) |
| *Zephyranthes bifida* | SRR3184143 | Garcia et al. (2014) | Meerow 3102 (FTG) |
| *Zephyranthes blumenavia* | SRR3183496 | Garcia et al. (2014) | Meerow-3115 (NA) |
| *Zephyranthes candida* | MW406476 | N/A | (HEAC) |
| *Zephyranthes cisandina* | SRR3183838 | Garcia et al. (2014) | N. García 2811/G23 (SGO) |
| *Zephyranthes flavissima* | SRR3185396 | Garcia et al. (2014) | Meerow 2418 (FTG) |
| *Zephyranthes graciliflora* | SRR3189437 | Garcia et al. (2014) | Leuenberger 4808g (B) |
| *Zephyranthes graciliflora* | SRR3185213 | Garcia et al. (2014) | Leuenberger 2811 (B) |
| *Zephyranthes jamesonii* | SRR3189443 | Garcia et al. (2014) | Leuenberger 6960 (B) |
| *Zephyranthes martinezii* | SRR3189195 | Garcia et al. (2014) | Meerow 2437 (FTG) |
| *Zephyranthes mesochloa* | SRR3185397 | Garcia et al. (2014) | Meerow 2420 (FTG) |
| *Zephyranthes montana* | SRR3184083 | Garcia et al. (2014) | N. García 243/G53 (FLAS) |
| *Zephyranthes phycelloides* | MW348956 | Contreras-Díaz et al. (2022) | EIF14539 (UOA) |
| *Zephyranthes* sp. | SRR3189190 | Garcia et al. (2014) | Dutilh sn (UEC) |
