# Supplementary material for: The origin and diversification of Amaryllidaceae: A phylogenetic and biogeographic analysis
Source: Am J Bot. 2025 Sep 11;112(9):e70092. doi: 10.1002/ajb2.70092 (PMC12464465; doi:10.1002/ajb2.70092)
Supplement: Supplementary file 1 — Appendix S1. Amaryllidoideae tribes groups used in this study. Appendix S2. List of new plastomes constructed for this study, including voucher information, GenBank accessions, and length of the whole plastome. Appendix S3. List of taxa acquired from previous publications, including GenBank or SRA accessions and citation information. Appendix S4. Taxa used as starting seeds for GetOrganelle assemblies of the SRA data used. Appendix S5. Asparagales taxa used to place fossils and secondary dates for the divergence analysis, with source and collection vouchers. Appendix S6. Taxa included in the wider Asparagales dated phylogeny. Appendix S7. Biogeographic areas assigned using the World Geographical Scheme for Recording Plant Distributions. Appendix S8. Maximum likelihood phylogeny of Amaryllidaceae based on 78 plastid protein‐coding genes. Appendix S9. Maximum likelihood consensus phylogeny of Amaryllidaceae based on 75–78 plastid protein‐coding genes. Appendix S10. Bayesian inference consensus phylogeny of Amaryllidaceae based on 78 plastid protein‐coding genes. Appendix S11. Tanglegram between plastome maximum likelihood and Bayesian inference phylogenies of the American clade showing incongruence between the two analyses. Appendix S12. AICc statistic scores for BioGeoBEARS biogeographic analysis conducted using RASP version 4.2. Appendix S13. List of the four most probable reconstructed ancestral origins for Amaryllidaceae, all subfamilies, and key groups. [file AJB2-112-e70092-s001.zip › Appendix_S5.docx]

**Appendix S5** – Asparagales taxa used to place fossils and secondary dates for the divergence analysis, with source and collection vouchers. Sources: 1 = Givnish et al. (2018), 2 = McKain et al., (2016), 3 = Sheng et al. (2017), 4 = Pfanzelt et al. (2019), 5 = Amor et al. (2020), 6 = Steele et al., (2017), 7 = Smissen & Scheele (2022), 8 = Lin et al. (2015), 9 = Out et al. (2020), 10 = Lee et al. (2019), 11 = Givnish et al. 2010, 12 = Kim et al. (2015), 13 = Leebens-Mack et al. (2005), 14 = Janssen et al. (2007). Herbarium codes: CHR = Manaaki Whenua - Landcare Research, CONC = Herbario Universidad de Concepción, FLAS = Florida Museum of Natural History, GA = University of Georgia, HAST = Academia Sinica Taiwan Taipei, K = Royal Botanic Gardens Kew, KRIB = Korea Research Institute of Bioscience and Biotechnology, LDU = Herbarium of Longdong University Gansu, RBGV = Royal Botanic Gardens Victoria.

| **Taxon** | **GenBank Accessions** | **Sources** | **Voucher** |
| --- | --- | --- | --- |
| *Agave attenuata* | KX931447 | 1,2 | McKain 109 (GA) |
| *Asparagus officinalis* | KY364194 | 3 | Leebens-Mack 1001-2010 (GA) |
| *Astelia australiana* | MH752984 | 4 | 13384 (RBGV) |
| *Astelia pumila* | MN839533 | 5 | 181151 S. Pfanzelt 539 (CONC) |
| *Cordyline australis* | JQ274066 | 1,6 | Hudson Seeds, La Honda, CA |
| *Cordyline indivisa* | KX822776 | N/A | 20071291 (K) |
| *Dianella nigra* | MN239902 | 7 | 611038 (CHR) |
| *Goodyera fumata* | KJ501999 | 8 | 139294 (HAST) |
| *Hemerocallis citrina* | MN872235 | 9 | XB20190913 (LDU) |
| *Hemerocallis fulva* | MT806177 | 10 | 37°05'40.4"N 127°24'23.7"E (KRIB) |
| *Nolina atopocarpa* | HQ183616; KX931462 | 1,2,11 | McKain 114 (GA) |
| *Ophiopogon japonicus* | JQ274072 | 1,6 | Steele 1090 |
| *Phragmipedium longifolium* | KM032625 | 1,12 | Whitten 2804 (FLAS) |
| *Ruscus aculeatus* | JQ274073 | 1,6 | B&T World Seeds, France |
| *Xanthorrhoea preissii* | JQ274082 | 1,6 | Pires 014-4 June 2010 |
| *Xanthorrhoea preissii* | KX822774 | N/A | 20005062 (K) |
| *Yucca brevifolia* | KX931466 | 2 | Smith 2010 |
| *Yucca schidigera* | DQ069546; EU016685 | 1, 13,14 | Leebens-Mack 2005 (GA) |
