# Supplementary material for: The origin and diversification of Amaryllidaceae: A phylogenetic and biogeographic analysis
Source: Am J Bot. 2025 Sep 11;112(9):e70092. doi: 10.1002/ajb2.70092 (PMC12464465; doi:10.1002/ajb2.70092)
Supplement: Supplementary file 1 — Appendix S1. Amaryllidoideae tribes groups used in this study. Appendix S2. List of new plastomes constructed for this study, including voucher information, GenBank accessions, and length of the whole plastome. Appendix S3. List of taxa acquired from previous publications, including GenBank or SRA accessions and citation information. Appendix S4. Taxa used as starting seeds for GetOrganelle assemblies of the SRA data used. Appendix S5. Asparagales taxa used to place fossils and secondary dates for the divergence analysis, with source and collection vouchers. Appendix S6. Taxa included in the wider Asparagales dated phylogeny. Appendix S7. Biogeographic areas assigned using the World Geographical Scheme for Recording Plant Distributions. Appendix S8. Maximum likelihood phylogeny of Amaryllidaceae based on 78 plastid protein‐coding genes. Appendix S9. Maximum likelihood consensus phylogeny of Amaryllidaceae based on 75–78 plastid protein‐coding genes. Appendix S10. Bayesian inference consensus phylogeny of Amaryllidaceae based on 78 plastid protein‐coding genes. Appendix S11. Tanglegram between plastome maximum likelihood and Bayesian inference phylogenies of the American clade showing incongruence between the two analyses. Appendix S12. AICc statistic scores for BioGeoBEARS biogeographic analysis conducted using RASP version 4.2. Appendix S13. List of the four most probable reconstructed ancestral origins for Amaryllidaceae, all subfamilies, and key groups. [file AJB2-112-e70092-s001.zip › Appendix_S6.docx]

**Appendix S6** – Taxa included in the wider Asparagales dated phylogeny.

| **Taxon** | **Accession** |
| --- | --- |
| *Acis autumnalis* | PP853182 |
| *Acis rosea* | PP853187 |
| *Agapanthus caulescens* | PP853183 |
| *Agapanthus praecox*subsp.*orientalis* | PP853184 |
| *Agave attenuata* | KX931447 |
| *Allium cernuum* | MT348440 |
| *Allium ochotense* | MT348451 |
| *Allium senescens* | MT348450 |
| *Amaryllis belladonna* | MZ433380 |
| *Ammocharis coranica* | PP853186 |
| *Asparagus officinalis* | KY364194 |
| *Astelia australiana* | MH752984 |
| *Astelia pumila* | MN839533 |
| *Boophone disticha* | PP853190 |
| *Boophone haemanthoides* | PP853189 |
| *Brunsvigia bosmaniae* | PP853191 |
| *Brunsvigia josephinae* | PP853193 |
| *Calostemma purpureum* | MT348445 |
| *Clinanthus variegatus* | PP853199 |
| *Clivia caulescens* | PP853196 |
| *Clivia gardenii* | PP853197 |
| *Cordyline australis* | JQ274066 |
| *Cordyline indivisa* | KX822776 |
| *Crinum asiaticum* | PP853198 |
| *Crinum jagus* | PP853195 |
| *Cyrtanthus falcatus* | PP853200 |
| *Cyrtanthus sanguineus* | PQ684178-PQ684254 |
| *Dianella nigra* | MN239902 |
| *Galanthus elwesii* | PP853202 |
| *Galanthus woronowii* | PP853204 |
| *Gethyllis* cf. *villosa* | PP853205 |
| *Gilliesia graminea* | MT348447 |
| *Goodyera fumata* | KJ501999 |
| *Haemanthus albiflos* | PP853209 |
| *Haemanthus coccineus* | PP853206 |
| *Hannonia hesperidium* | PP853207 |
| *Hemerocallis citrina* | MN872235 |
| *Hemerocallis fulva* | MT806177 |
| *Hessea breviflora* | PP853210 |
| *Hessea pulcherrima* | PP853212 |
| *Hieronymiella argentina* | PP853213 |
| *Hippeastrum reginae* | MT701522 |
| *Hippeastrum reticulatum* | MT701523 |
| *Hippeastrum striatum* | MT133568 |
| *Hymenocallis speciosa* | PP853214 |
| *Ipheion sellowianum* | PP853216 |
| *Ismene* × *deflexa* | PP853215 |
| *Lapiedra martinezii* | MN539612 |
| *Leucocoryne ixioides* | PP853218 |
| *Leucocoryne purpurea* | PP853219 |
| *Leucojum aestivum* | PP853217 |
| *Lycoris aurea* | PP853220 |
| *Lycoris chinensis* | MT700549 |
| *Narcissus asturiensis* | PP853188 |
| *Narcissus panizzianus* | PP853229 |
| *Narcissus poeticus* | MH706763 |
| *Nerine appendiculata* | PP853222 |
| *Nerine bowdenii* | PP853223 |
| *Nerine humilis* | PP853228 |
| *Nerine krigei* | PP853224 |
| *Nerine platypetala* | PP853226 |
| *Nerine rehmannii* | PP853227 |
| *Nerine undulata* | PQ684107-PQ684117 |
| *Nolina atopocarpa* | HQ183616 |
| *Nolina atopocarpa* | KX931462 |
| *Nothoscordum bivalve* | MZ019481 |
| *Ophiopogon japonicus* | JQ274072 |
| *Pancratium canariense* | PP853230 |
| *Pancratium maritimum* | MHN539614 |
| *Paramongaia weberbaueri* | PP853231 |
| *Phragmipedium longifolium* | KM032625 |
| *Proiphys amboinensis* | PP853232 |
| *Ruscus aculeatus* | JQ274073 |
| *Scadoxus* cf. *multiflorus* | PP853233 |
| *Stenomesson miniatum* | PP853236 |
| *Sternbergia candida* | PP853234 |
| *Sternbergia lutea* | PP853235 |
| *Strumaria barbarae* | PP853237 |
| *Strumaria chaplinii/discifera* | PP853238 |
| *Strumaria gemmata* | PP853239 |
| *Strumaria phonolithica* | PP853240 |
| *Strumaria picta* | PP853241 |
| *Strumaria tenella* subsp. *orientalis* | PP853242 |
| *Strumaria truncata* | MN539615 |
| *Tulbaghia violacea* | PQ684037-PQ684106 |
| *Urceolina amazonica* | PP853201 |
| *Urceolina subedentata* | PP853194 |
| *Vagaria parviflora* | PP853243 |
| *Worsleya procera* | PP853244 |
| *Xanthorrhoea preissii* | JQ274082 |
| *Xanthorrhoea preissii* | KX822774 |
| *Yucca brevifloia* | KX931466 |
| *Yucca schidigera* | DQ069546 |
| *Yucca schidigera* | EU016685 |
| *Zephyranthes candida* | MW406476 |
| *Zephyranthes phycelloides* | MW348956 |
