# Supplementary material for: The origin and diversification of Amaryllidaceae: A phylogenetic and biogeographic analysis
Source: Am J Bot. 2025 Sep 11;112(9):e70092. doi: 10.1002/ajb2.70092 (PMC12464465; doi:10.1002/ajb2.70092)
Supplement: Supplementary file 1 — Appendix S1. Amaryllidoideae tribes groups used in this study. Appendix S2. List of new plastomes constructed for this study, including voucher information, GenBank accessions, and length of the whole plastome. Appendix S3. List of taxa acquired from previous publications, including GenBank or SRA accessions and citation information. Appendix S4. Taxa used as starting seeds for GetOrganelle assemblies of the SRA data used. Appendix S5. Asparagales taxa used to place fossils and secondary dates for the divergence analysis, with source and collection vouchers. Appendix S6. Taxa included in the wider Asparagales dated phylogeny. Appendix S7. Biogeographic areas assigned using the World Geographical Scheme for Recording Plant Distributions. Appendix S8. Maximum likelihood phylogeny of Amaryllidaceae based on 78 plastid protein‐coding genes. Appendix S9. Maximum likelihood consensus phylogeny of Amaryllidaceae based on 75–78 plastid protein‐coding genes. Appendix S10. Bayesian inference consensus phylogeny of Amaryllidaceae based on 78 plastid protein‐coding genes. Appendix S11. Tanglegram between plastome maximum likelihood and Bayesian inference phylogenies of the American clade showing incongruence between the two analyses. Appendix S12. AICc statistic scores for BioGeoBEARS biogeographic analysis conducted using RASP version 4.2. Appendix S13. List of the four most probable reconstructed ancestral origins for Amaryllidaceae, all subfamilies, and key groups. [file AJB2-112-e70092-s001.zip › Appendix_S12.docx]

**Appendix S12** – AICc statistic scores for BioGeoBEARS biogeographical analysis conducted using RASP v.4.2. AICc weighted (AICc_wt) used to select best model.

| **Model Test** | **LnL** | **numparams** | **d** | **e** | **j** | **AICc** | **AICc_wt** |
| --- | --- | --- | --- | --- | --- | --- | --- |
| DEC | -242.5 | 2 | 0.0046 | 0.039 | 0.00000 | 489.2 | 1.10E-10 |
| DEC+J | -242.3 | 3 | 0.0039 | 0.027 | 0.00390 | 490.9 | 4.90E-11 |
| DIVALIKE | -249.2 | 2 | 0.0056 | 0.06 | 0.00000 | 502.5 | 1.40E-13 |
| DIVALIKE+J | -249.0 | 3 | 0.0059 | 0.068 | 0.00001 | 504.4 | 5.60E-14 |
| BAYAREALIKE | -221.6 | 2 | 0.0028 | 0.051 | 0.00000 | 447.5 | 0.13 |
| BAYAREALIKE+J | -218.6 | 3 | 0.002 | 0.048 | 0.00380 | 443.7 | 0.87 |

| **alt** | **null** | **LnL**  **alt** | **LnL**  **null** | **DF**  **alt** | **DF**  **null** | **DF** | **Dstat** | **Pval** | **Test** | **AIC 1** | **AIC 2** | **AICwt 1** | **AICwt 2** | **AICwt ratio model 1** | **AICwt ratio model 2** |
| --- | --- | --- | --- | --- | --- | --- | --- | --- | --- | --- | --- | --- | --- | --- | --- |
| DEC+J | DEC | -242.3 | -242.5 | 3 | 2 | 1 | 0.51 | 0.480 | chi-squared | 490.5 | 489.0 | 0.3 | 0.68 | 0.47 | 2.11 |
| DIVALIKE+J | DIVALIKE | -249.0 | -249.2 | 3 | 2 | 1 | 0.30 | 0.580 | chi-squared | 504.0 | 502.3 | 0.3 | 0.70 | 0.43 | 2.34 |
| BAYAREALIKE+J | BAYAREALIKE | -218.6 | -221.6 | 3 | 2 | 1 | 5.98 | 0.014 | chi-squared | 443.3 | 447.3 | 0.9 | 0.12 | 7.31 | 0.14 |
